# Network Pharmacology Deciphers the Action of Bioactive Polypeptide in Attenuating Inflammatory Osteolysis via the Suppression of Oxidative Stress and Restoration of Bone Remodeling Balance

**DOI:** 10.1155/2022/4913534

**Published:** 2022-04-14

**Authors:** Zichen Cui, Changgong Feng, Jiazheng Chen, Yi Wang, Qi Meng, Shihao Zhao, Yuanji Zhang, Dianjie Feng, Ziqing Li, Shui Sun

**Affiliations:** ^1^Department of Joint Surgery, Shandong Provincial Hospital Affiliated to Shandong First Medical University, Jinan, Shandong 250021, China; ^2^Orthopaedic Research Laboratory, Medical Science and Technology Innovation Center, Shandong First Medical University & Shandong Academy of Medical Sciences, Jinan, Shandong 250117, China; ^3^Department of Joint Surgery, Shandong Provincial Hospital, Shandong University, Jinan, Shandong 250012, China

## Abstract

Oxidative stress involves enormously in the development of chronic inflammatory bone disease, wherein the overproduction of reactive oxygen species (ROS) negatively impacts the bone remodeling via promoting osteoclastogenesis and inhibiting osteogenesis. Lacking effective therapies highlights the importance of finding novel treatments. Our previous study screened a novel bioactive peptide D7 and demonstrated it could enhance the cell behaviors and protect bone marrow mesenchymal stem cells (BMSCs). Since BMSCs are progenitor cells of osteoblast (OB), we therefore ask whether D7 could also protect against the progress of inflammatory osteolysis. To validate our hypothesis and elucidate the underlying mechanisms, we first performed network pharmacology-based analysis according to the molecule structure of D7, and then followed by pharmacological evaluation on D7 by in vitro lipopolysaccharide(LPS)-induced models. The result from network pharmacology identified 20 candidate targets of D7 for inflammatory osteolysis intervention. The further analysis of Gene Ontology (GO)/KEGG pathway enrichment suggested the therapeutic effect of D7 may primarily affect osteoclast (OC) differentiation and function during the inflammatory osteolysis. Through validating the real effects of D7 on OC and OB as postulated, results demonstrated suppressive effects of D7 on LPS-stimulated OC differentiation and resorption, via the inhibition on OC marker genes. Contrarily, by improving the expression of OB marker genes, D7 displayed promotive effects on OB differentiation and alleviated LPS-induced osteogenic damage. Further mechanism study revealed that D7 could reduce LPS-induced ROS formation and strengthen antioxidants expressions in both OC and OB precursors, ameliorating LPS-triggered redox imbalance in bone remodeling. Taken together, our findings unveiled therapeutic effects of D7 against LPS-induced inflammatory osteolysis through the suppression of oxidative stress and the restoration of the bone remodeling process, providing a new therapeutic candidate for chronic inflammatory bone diseases.

## 1. Introduction

Chronic inflammatory bone diseases, such as rheumatoid arthritis, are mainly characterized by serious destruction of the bone tissue [1, 2]. Lacking safe, effective, and affordable therapies highlights the importance of identifying crucial determinants in the process of inflammatory osteolysis [3]. Mature osteoclasts (OCs) are crucial effector cells for bone erosion during inflammatory bone loss [4]. The hyper-activation of OCs due to inflammatory state adversely affects bone homeostasis and finally leads to the destruction of bone remodeling balance [5]. On the other hand, inflammation-induced apoptosis and differentiation failure of osteoblasts (OBs) also contribute to the imbalance of the bone remodeling process, and accelerate the inflammatory osteolysis [6]. Therefore, uncovering new avenues to target inflammatory osteolysis by inhibiting bone-degrading OCs and enhancing bone-forming OBs on the same page is vital towards treating these debilitating bone diseases.

Under the stimulation of inflammatory factors such as lipopolysaccharide (LPS), oxidative stress involves in the overproduction of reactive oxygen species (ROS), therefore disrupting the redox balance of OCs and OBs, and eventually leading to imbalanced bone remodeling [7, 8]. Although ROS at physiological levels helps maintain redox signaling in cells via promoting different post-translational modification of protein kinases and phosphatases, excessive ROS contributes to different cell fates of OCs and OBs [9–11]. By activating the differentiation of OC precursors via the promotion of critical functional genes such as nuclear factor of activated T-cells 1 (Nfatc1) and TNF receptor-associated factor 6 (Traf6) and enhanced the level of tartrate-resistant acid phosphatase (TRAP) [8], overproduction of ROS leads to an increase in OCs number and strengthens the bone resorption activity via secreting acid and collagenase, such as cathepsin K (Ctsk) [12–14]. Concurrently, elevated formation of ROS causes osteogenic damage, which in turn leads to fatal injury and programmed cell death of OBs [15], including impairments on proliferation and differentiation of OBs due to reduced expression of critical osteogenic genes such as alkaline phosphatase (ALP), runt-related transcription factor 2 (Runx2), and type I collagen alpha 1 chain (Col1*α*1). On the contrary, the application of antioxidants, acting as direct scavengers of ROS, could promote the differentiation and mineralization of OBs, as well as dwindle the activity of OCs through confronting oxidative stress [16–18]. Therefore, either reducing ROS production or increasing the level of antioxidants would be an effective way to target inflammatory osteolysis via the correction of the oxidative stress-induced imbalance of bone remodeling.

In recent years, bioactive peptides increasingly emerge their importance as the secondary structure of proteins, and have shown positive effects for human health including antihypertensive, antioxidation, and antimicrobial [19]. From our previous works, a bioactive cyclic polypeptide with specific amino acid sequence, called D7, had been stood out after 3 rounds of biopanning via phage display technology from Ph.D.C7C™ Phage Display Library [20]. D7 showed an excellent affinity to bone marrow mesenchymal stem cells (BMSCs) because of the cyclic structure formed by the disulfide linkage compared with linear peptides [20]. We further demonstrated that D7 could improve the adhesion, expansion and proliferation of BMSCs on *β*-tricalcium phosphate scaffolds which are commonly used for therapeutics of osteonecrosis of the femoral head (ONFH) [21]. Since activation of oxidative stress is a well-known cause of ONFH pathogenesis, and activators of ROS have been applied for the establishment of ONFH models in basic research, we therefore believed that the promotive effect of D7 on BMSCs may result from the confrontation on oxidative stress [13, 22]. By the same token, D7 may work on others bone cells participating in destructive bone diseases, such as hematopoietic stem cell- (HSCs)-derived OCs. HSCs and BMSCs are two major stem cells that exist in the bone marrow microenvironment and are critical for the regulation of bone remodeling. Additionally, the process of OCs differentiation from HSCs is tightly affected by ROS production, which is a similar step that D7 involved in BMSCs against oxidative stress [10]. Therefore, we believe that D7 may also have a role in affecting HSCs-derived OCs during the development of ONFH.

Following the expeditious development of bioinformatics databases, a state-of-the-art technique, named network pharmacology, has been widely used in expanding the application scope of newly emerged drugs or elucidating the working mechanisms of complicated drugs, from the molecular level to disease level [23]. Based on huge databases, network pharmacology updates the traditional research mode of “one target, one drug” mode to a detailed “drug-target, target-pathway networks.”

Hence, in the present study, we used network pharmacology-related databases to determine the pharmacological network of D7 on inflammatory osteolysis, and predict potential gene targets and pathways. Further, experiments were conducted based on the preliminary clues to validate the real effects of D7 on OCs and OBs, which are the crucial determinants in the process of inflammatory osteolysis, so that to provide a new candidate for the treatment of chronic inflammatory bone diseases.

## 2. Methods and Materials

### 2.1. Analysis of Overlapping Targets of D7 in Inflammatory Osteolysis

The predicted targets were extracted from the Swiss Target Prediction database (http://www.swisstargetprediction.ch/) [24] according to the structure of D7. Related inflammatory osteolysis targets were collected from the Gene-Cards database (https://www.genecards.org/) [25] using “Inflammatory osteolysis” as keywords. Then, the putative targets of D7 mapped to the inflammatory osteolysis-related targets to acquire overlapping targets. A Venn diagram was obtained with the Venn diagram website (http://bioinformatics.psb.ugent.be/webtools/Venn/) [26] to identify the intersection of D7 and inflammatory osteolysis [27].

### 2.2. Network Analysis and Functional Annotation for Targets Related to Inflammatory Osteolysis of D7

String database (https://string-db.org/cgi/input.pl) was used to construct a PPI network with the overlapping targets of D7 in inflammatory osteolysis. The edges indicated both functional and physical protein associations, and the line color indicated the type of interaction evidence. The minimum required interaction score was low confidence (0.15).

The common targets of D7 and inflammatory osteolysis were imported into the Functional Annotation tool of Database for Annotation, Visualization and Integrated Discovery (DAVID) 6.8 (https://david.ncifcrf.gov/) [28] to perform GO biological process (BP), molecular function (MF), cell component (CC), and KEGG pathway analyses. Screening the items with correction *p* ≤ 0.01, the top ten pathways with the most enriched targets were selected to visualize the data by Graphpad.

### 2.3. Reagents and Antibodies

Cell culture reagents, including fetal bovine serum (FBS; 10099141C), minimum essential medium *α* (*α*-MEM; C12571500BT), and penicillin/streptomycin (P/S; 15140122), were all purchased from Gibco (USA). Reagents for cell differentiation and stimulation included lipopolysaccharide (L8274; Sigma-Aldrich), cyclic polypeptide D7 (Scilight-Peptide Inc.), ascorbic acid (A8960; Sigma-Aldrich), *β*-glycerophosphate disodium (G9422; Sigma-Aldrich), dexamethasone (HY-14648; MCE), macrophage colony-stimulating factor (M-CSF; 416-ML-050; R&D Systems), and receptor activator of nuclear factor-*κ*B ligand (RANKL; 462-TEC-010; R&D Systems). Primary antibodies that used in western blotting (WB) for the detection of ALP (ab229126), Runx2 (ab236639), and Ctsk (ab187647) were from Abcam; for Col1*α*1 (72026t) detection was from Cell Signaling Technology; for Nfatc1 (sc-7294) and Traf6 (sc-8409) detections were from Santa Cruz; for *β*-actin (hrp-66009) detection was from Proteintech. Secondary antibodies were purchased from Proteintech, including anti-rabbit (SA00001-2) and anti-mouse (SA00001-1).

### 2.4. Cell Culture and Differentiation

In brief, bone marrow-derived monocytes (BMMs) were isolated from 8 to 10 weeks old C57BL/6 male mice. Bone marrow cells flushed out from long bones were incubated in a complete medium (CM; *α*-MEM with 1% P/S and 10% FBS) overnight at 37°C. After harvesting the nonadherent cells, red blood cell lysate (BL503A; Biosharp) was added for 3 min. 2 × 10^5^/ml cells were cultured in CM added M-CSF (25 ng/ml) for 3 days. Whereafter, adherent cells were then induced with M-CSF (25 ng/ml) and RANKL (40 ng/ml) in CM for 5 days differentiation or stimulated with M-CSF and RANKL for a 3-day OC pretreatment first to generate OC precursors and then changed to M-CSF and LPS for the remaining differentiation time [5].

Osteoblast precursor cells (OPCs) were extracted from postnatal day 5 C57BL/6 mice and isolated from the calvaria. The calvaria was incubated in collagenase solution which was *α*-MEM containing collagenase II (0.5 mg/ml) (17101015; Gibco) and 0.05% trypsin (15090046; Gibco) for 20 min at 37°C. After the new collagenase solution was replaced, the calvaria was cut into small fragments and cultured for another 20 min at 37°C, followed by the addition of *α*-MEM with 1% P/S and 15% FBS overnight. Then, the medium was switched to CM. Cells from the second to the fourth generation will be used in osteogenesis-induced. 5 × 10^4^/ml cells were incubated in plates and osteogenic induction medium (OIM; CM with 50 ug/ml ascorbic acid, 100 nM dexamethasone, and 10 mM *β*-glycerophosphate disodium salt hydrate) with or without 100 ng/ml LPS was replaced once the cells reached 80% confluency in plates. We replaced the medium every two days [29].

### 2.5. Peptide Affinity Assay

BMMs and OPCs were seeded in 24-well plates. BMMs were incubated in CM with 25 ng/ml M-CSF and 40 ng/ml RANKL, whereas OPCs were cultured with OIM, and then 20 *μ*M FITC-labeled D7 was added in each well at 37°C for 24 h. The BMMs and OPCs were fixed with 4% paraformaldehyde (PFA, BL539A; Biosharp) at room temperature for 20 min. After that, phalloidin-iFluor 594 (ab176757; Abcam) was subjected to stain the cells for 45 min in the dark. Then, 4′,6-diamidino-2-phenylindole (DAPI, C0065; Solarbio) was used to counterstain the cells at room temperature for 5 min. The cells were finally observed using an Image-Xpress Micro Confocal system (Molecular Devices) [20].

### 2.6. Cell Viability Assay

BMMs and OPCs were cultured in 96-well plates, and were evaluated for the D7-induced proliferation via a Cell Counting Kit-8 (HY-K0301; MCE) assay. BMMs were stimulated with different dosages of D7 (0, 10, 20, 50, and 100 *μ*M) in CM containing 25 ng/ml M-CSF with or without 40 ng/ml RANKL for 48 h, whereas OPCs were stimulated for 48 h in CM or OIM with different dosages of D7 (0, 10, 20, 50, 100, and 200 *μ*M). After adding CCK-8 to each well accordingly, cells were incubated at 37°C for 2 h, followed by absorbance measurement via a spectrophotometer (Multiskan GO 1510; ThermoFisher Scientific) at 450 nm.

### 2.7. TRAP Staining and Bone Resorption Assay

After the formation of mature OCs, PFA was used to fix cells for 20 min. Then following the manufacturer's instructions, cells were stained by using tartrate-resistant alkaline phosphatase kit (TRAP; 387A; Sigma-Aldrich). Cells that were TRAP-positive and contained three or more nuclei (multinucleated cells) were defined as OCs and counted by using a light microscope (IX53; Olympus) [30].

For bone resorption assay, cells were cultured in Osteo Assay Surface plates (3988; Corning). After the formation of mature OCs, cells were given an extra 2 days culture, followed by 5 min incubation of 10% bleach solution to remove all cells. After the plates were washed by deionized water and completely air-dried, bone resorption pits were observed by using a light microscope, and resorption areas were evaluated by the ImageJ software [30].

### 2.8. F-Actin Staining

Cells were fixed in PFA for 20 min after mature OCs were formed. The cells were then stained using phalloidin-iflour 594 at room temperature for 45 min in the dark, and the nuclei were then counterstained with DAPI for 5 min. The structures of actin rings were imaged using a fluorescence microscope (Axio Observer 3; Carl Zeiss) and analyzed by ImageJ software [30].

### 2.9. Acridine Orange (AO) Staining

After mature OCs generated in 24-well plates, the differentiated cells were incubated with AO (A6014; Sigma-Aldrich) solution diluted by *α*-MEM (10 *μ*g/ml) at 37°C for 15 min. After being washed three times with *α*-MEM, the acid vesicles of OCs were imaged under a fluorescence microscope. We used the same way on OCs in 96-well plates to detect the quantitative experiment of acid vesicles. The ratio of the absorbance at 652 nm (red light) and 485 nm (green light) was measured using a microplate reader [31].

### 2.10. ALP Staining and ARS Staining

OPCs were cultured in 24-well plates for 5 days treated with or without D7. After fixed with PFA for 20 min, cells were subjected to BCIP/NBT alkaline phosphatase color development kit (ALP; C3206; Beyotime). ALP staining is according to the instructions at room temperature for 10 min in the darkness.

OPCs were seeded in 24-well plates for 21 days treated with or without D7. Cells were stained with alizarin red S solution (1%, pH 4.2) (ARS; G1452; Solarbio) for 10 min at room temperature after fixed for 20 min with PFA [32, 33].

### 2.11. Western Blot Analysis

The cells were lysed using RIPA buffer (R0020; Solarbio) with phosphatase inhibitors (CW2383; Cwbio) and protease inhibitor (CW2200; Cwbio). Proteins extracted from each sample were determined using bicinchoninic acid protein assay kit (PC0020; Solarbio). Heat-denatured proteins were separated by 10% SDS-PAGE and transferred onto a 0.22 *μ*m polyvinylidene difluoride membrane (IPVH00010; Millipore). The membranes were blocked in 5% skim milk at room temperature for 1 h; then, each membrane was incubated with antibodies against Traf6, Nfatc1, Ctsk, Runx2, ALP, Col1*α*1, and HRP-*β*-actin at 4°C overnight. After the membranes were incubated with the respective secondary antibodies for 1 h at room temperature, the blots were detected using a chemiluminescent HRP substrate (wbkls0500; Millipore). We used the ImageJ software to quantify the grayscale value of bands.

### 2.12. Reverse Transcription Quantitative Polymerase Chain Reaction (RT-qPCR)

After the indicated treatments, total RNA was extracted using RNAiso Plus (9109; Takara) according to the manufacturer's protocol. Total RNA was converted to cDNA using a PrimeScript™ RT Reagent Kit (RR047A; Takara). The cDNA was amplified using the SYBR Green dye (AG11701; Accurate Biology) and quantified by a Roche LightCycler 480II (Roche, Basel, Switzerland). The genes we analyzed *ALP*, *Runx2*, *Col1α1*, *Traf6*, *Nfatc1*, and *Ctsk* were used the *β-actin* as endogenous control, whereas the mRNA levels of expression of *Cu/Zn-superoxide dismutase* (*Sod*), *Mn-Sod*, *glutathione reductase* (*Gr*), *glutathione peroxidase* (*Gpx*), and *Catalase* (*Cat*) were normalized to *glyceraldehyde 3-phosphate dehydrogenase* (*GAPDH*) mRNA level. All primer sequences we used are listed in Table 1. PCR amplification was performed using a Roche LightCycler 480II.

### 2.13. Intracellular ROS Measurement

The level of intracellular ROS was detected using a Reactive Oxygen Species Assay Kit (DCFH-DA; s0033; Beyotime). BMMs and OPCs were seeded in 6-well plates. BMMs were incubated in M-CSF and RANKL with or without D7 for 24 h, or stimulated with M-CSF and LPS with or without D7 for 24 h, whereas OPCs were incubated in OIM with or without D7 for 24 h, or stimulated with OIM and LPS with or without D7 for 24 h. Cells were cultured with DCFH-DA (10 *μ*M) diluted with serum-free *α*-MEM for 20 min at 37°C. After being washed three times with *α*-MEM, cells were observed by a Nikon fluorescence microscope, and fluorescence intensity was determined by the ImageJ software [34].

### 2.14. Statistical Analysis

Data are expressed as the mean ± standard deviation (SD). One-way ANOVA was conducted for multiple comparisons. A value of *p* < 0.05 was considered significant. Statistical analysis was performed using Graphpad software.

## 3. Results

### 3.1. Putative Targets and Biological Pathways of D7 on Inflammatory Osteolysis Prevention and Treatment

**Figure 1:**
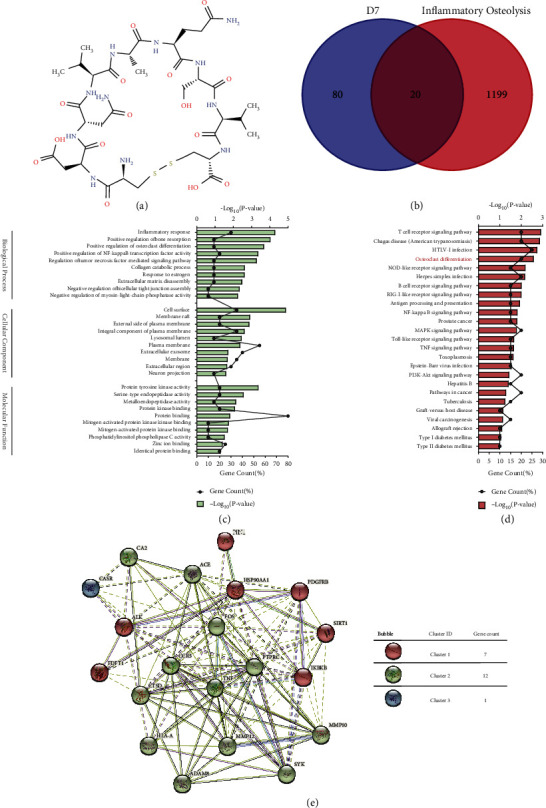
Putative targets and biological pathways of D7 on inflammatory osteolysis prevention and treatment. (a) The cyclic peptide D7 is a loop heptapeptide including seven amino acids (Asp-Asn-Val-Ala-Gln-Ser-Val) and two cysteine residues which contributed to form an intramolecular disulfide linkage. (b) Venn diagram summarized the number of candidate targets of D7 on inflammatory osteolysis. (c) Gene Ontology (GO) enrichment analysis to identify top 10 terms of biological characteristics, including biological process (BP), molecular function (MF), and cell component (CC), corresponding to the candidate targets. (d) KEGG pathway analysis suggested a close correlation between D7 and osteoclast (OC) differentiation. (e) D7-specific proteins interaction network and cluster annotation.

To characterize the molecular mechanism of D7 on preventing and treating inflammatory osteolysis, network pharmacology-based analysis was performed according to the molecule structure of D7 (Figure 1(a)). 100 putative targets were retrieved from the Swiss Target Prediction database (Supplement Table 1), and 1219 inflammatory osteolysis-related human genes were collected from the GeneCards database (Supplement Table 2). Then, these putative targets of D7 were mapped with inflammatory osteolysis-related human genes, wherein 20 targets of D7 were identified as the candidate targets for inflammatory osteolysis prevention and treatment (Figure 1(b)) (Table 2).

To further reveal the biological characteristics of these 20 putative targets of D7 on inflammatory osteolysis, GO and KEGG pathway enrichment analysis to identify biological processes and canonical pathways were conducted via the DAVID Bioinformatics Resources 6.8. Top 10 enriched terms that met the requirements of Count ≥ 2 and *p* value < 0.05 of statistical significance in BP, CC, and MF were shown in Figure 1(c). After comprehensively analyzing the terms based on the significance from both Gene Count and *p* value, the results indicate D7 may regulate OC differentiation and bone resorption via protein tyrosine kinase activity and endopeptidase activity, as well as protein binding and protein kinase binding in the cell surface, plasma membrane, and extracellular region to exert its effect on the inflammatory response. In addition, the pathway enrichment and proteins interaction network also suggested a close correlation between D7, inflammatory markers, and OC differentiation (Figures 1(d) and 1(e)), supporting the potential therapeutic effects of D7 on preventing and treating inflammatory osteolysis by primarily affecting OC differentiation and function.

### 3.2. D7 Maintains Its Bioactivity in BMMs and Impairs LPS-Stimulated Osteoclastogenesis

**Figure 2:**
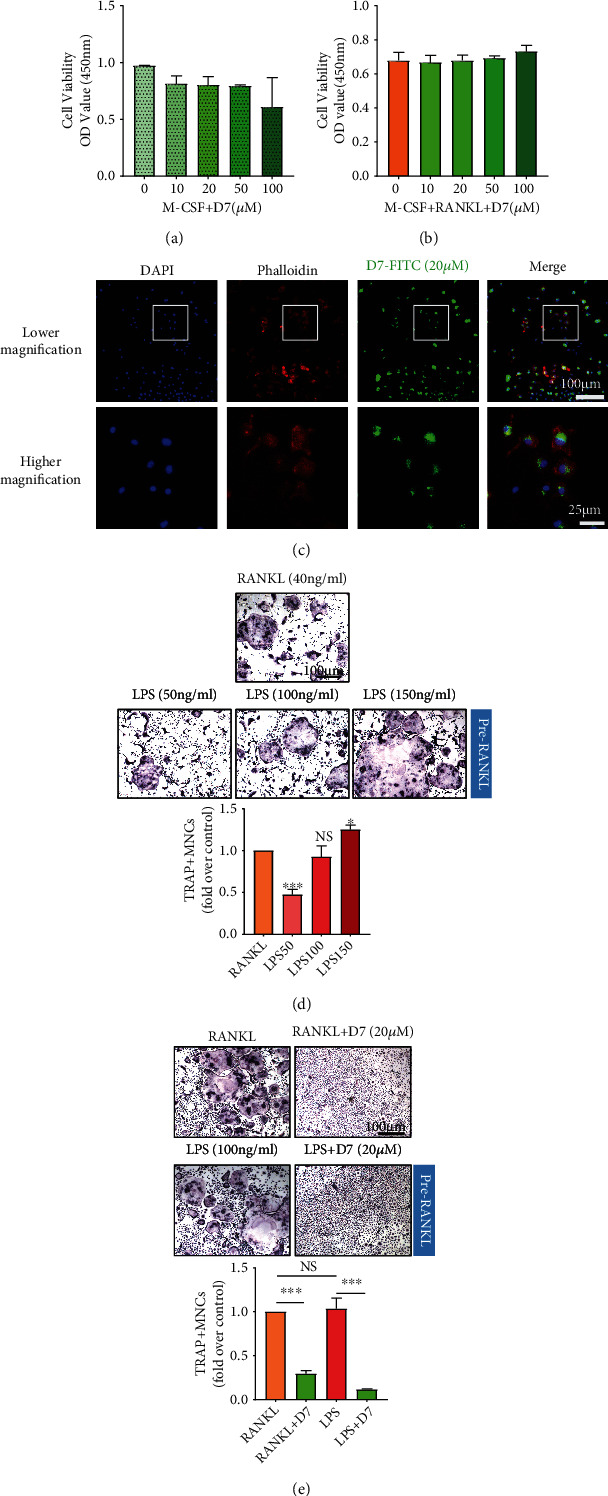
D7 maintains its bioactivity in BMMs and impairs LPS-stimulated osteoclastogenesis. (a) CCK-8 assay for cell viability test. Bone marrow-derived monocytes (BMMs) were stimulated with D7 (0–100 *μ*M) in proliferating medium containing M-CSF (25 ng/ml) only or in (b) OC differentiation medium containing M-CSF and RANKL (40 ng/ml) for 48 h (*n* = 3). (c) Fluorescence staining demonstrated the affinity of D7 towards BMMs. The cells induced with FITC-labeled D7 (20 *μ*M) for 48 h were observed under a high-content microscope. The nuclei and cytoskeletons of the cells were counterstained with DAPI and phalloidin-iflour 594, respectively. Scale bar in lower magnification = 100 *μ*m, scale bar in higher magnification = 25 *μ*m. (d) TRAP staining of OCs differentiated from BMMs under different stimuli. BMMs were induced with M-CSF (25 ng/mL) and RANKL (40 ng/mL) for a 5-day OC full differentiation, or induced with M-CSF and RANKL for a 3-day OC pretreatment first to generate OC precursors and then changed to M-CSF and LPS (50, 100, and 150 ng/mL) for the remaining differentiation time. Quantitative analysis of TRAP-positive cells that contained more than three nuclei (MNCs) was shown in the lower panel, and results were presented as the means ± SD (*n* = 3). ∗*p* < 0.05, ∗∗∗*p* < 0.001, and NS (not statistically significant) versus (VS) the RANKL group. Scale bar = 200 *μ*m. (e) TRAP staining of OCs differentiated from BMMs under various treatments of D7. BMMs were induced with M-CSF (25 ng/mL) and RANKL (40 ng/mL) for a 5-day OC full differentiation with or without D7 (20 *μ*M), or stimulated with M-CSF and RANKL for a 3-day OC pretreatment first to generate OC precursors and then replaced to M-CSF and LPS (100 ng/mL) with or without D7 (20 *μ*M) for the remaining differentiation time. Scale bar = 200 *μ*m. TRAP-positive MNCs were counted as previously described (*n* = 3). ∗∗∗*p* < 0.001 and NS VS the indicated groups.

To validate the real effect of D7 on OC differentiation and function during inflammatory osteolysis as postulated, various assays were conducted accordingly. Via the CCK8 test, the cell viability of BMMs was negatively affected when the dosage of D7 was used up to 100 *μ*M in the presence of M-CSF (Figure 2(a)). However, when combining the addition of RANKL, the cell viability of BMMs showed slightly increased with the usage of D7, even under 100 *μ*M dosage (Figure 2(b)). But there was no statistical significance in CCK-8 assays (Figures 2(a) and 2(b)), indicating the dose of D7 below 100 *μ*M was safe to use on BMMs. Therefore, the intermediate concentration (20 *μ*M) was selected in order to set up a proper baseline to reflect up or down-effect on the cellular function in the subsequent experiment. We next verified the bioactivity of D7 in BMMs based on the cell viability test. BMMs were stimulated with 20 *μ*M FITC-labeled D7 in CM with M-CSF and RANKL for 48 h. The result showed that D7 displayed an excellent bioactivity in BMMs, and D7 was mostly presented in the cytoplasm of BMMs but not in the nucleus (Figure 2(c)), supporting the biological characteristics of CC postulated from network pharmacology analysis (Figure 1(c)).

Further, to define the effect of D7 on LPS-stimulated osteoclastogenesis, BMMs were induced with RANKL first for 72 h and then replaced with LPS alone according to previous studies [5]. TRAP staining showed that LPS promoted the formation of OCs in a dose-dependent manner, and 100 ng/ml LPS demonstrated the same promotive effect on osteoclastogenesis compared with pure RANKL stimulation (Figure 2(d)). With D7 (20 *μ*M) addition, the generation of OCs was significantly impaired no matter stimulated by RANKL or LPS (Figure 2(e)), indicating a strong suppressive effect of D7 on LPS-stimulated osteoclastogenesis.

### 3.3. D7 Suppresses the Resorption Function of OCs

**Figure 3:**
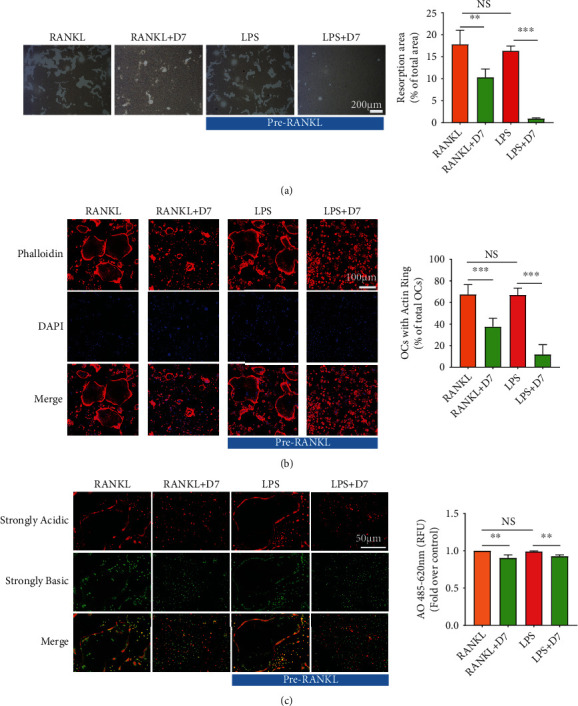
D7 suppresses the resorption function of OCs. (a) Bone resorption assay of OCs differentiated from BMMs under various treatments of D7. Cell treatments were described as previous, and the resorption area was measured by ImageJ software. The quantitative results were presented as the means ± SD (*n* = 3). ∗∗*p* < 0.01, ∗∗∗*p* < 0.0001, and NS VS the indicated groups. Scale bar = 200 *μ*m. (b) F-actin rings were visualized using a fluorescence microscope after the staining of phalloidin-iflour 594 and DAPI. The number of quantitative results was calculated by ImageJ software and presented as the means ± SD (*n* = 3). ∗∗∗*p* < 0.001 and NS VS the indicated groups. Scale bar = 100 *μ*m. (c) Acidified compartments in OCs were stained with acridine orange (AO) dye and imaged under a fluorescence microscope. Quantitative analysis of AO intensity was measured by a microplate reader at the absorbance of 485 nm and presented as means ± SD (*n* = 3). ∗∗*p* < 0.001 and NS VS the indicated groups. Scale bar = 50 *μ*m.

We next examine the impact of D7 on OCs function. Evidenced by the dramatic reduction in the area of resorption pits, D7 significantly hampered the bone resorption process, especially under LPS-stimulated conditions (Figure 3(a)), which is consistent with the previous analysis of network pharmacology and osteoclastogenesis study. Furthermore, we evaluated the influence of D7 on F-actin ring formation and osteoclastic acidification, which are prerequisites of bone resorption. Phalloidin-iflour 594 staining demonstrated 68% of RANKL-stimulated and 67% of LPS-stimulated OCs possessed intact actin rings, whereas, under D7 condition, the ring number substantially shrunk to 38% and 12%, respectively (Figure 3(b)). Meanwhile, AO staining showed less red fluorescence in OCs after D7 addition, reflecting less accumulation of acidified compartments in OCs treated with D7 (Figure 3(c)). Taken together, these findings provided a negative impact on LPS-stimulated OCs function due to D7 treatment.

### 3.4. D7 Inhibits the Expression of OC Marker Genes

**Figure 4:**
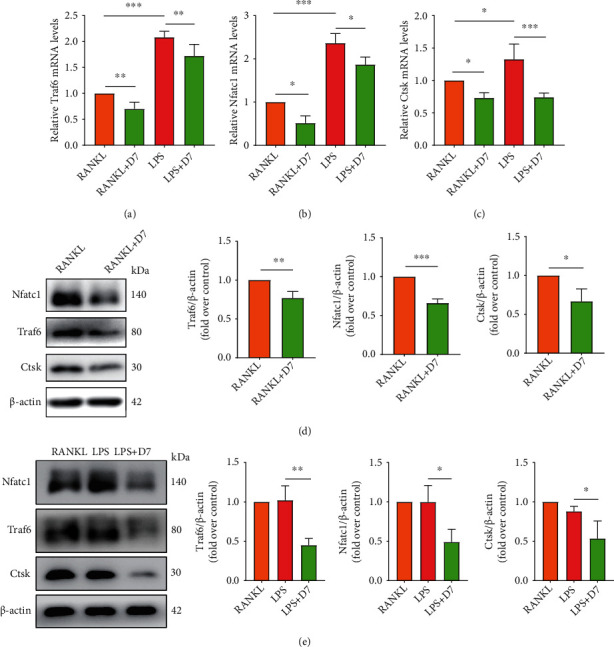
D7 inhibits the expression of OC marker genes. (a)–(c) RT-qPCR showed the mRNA levels of Traf6 (a), Nfatc1 (b), and Ctsk (c) in OCs after different treatments. Quantitative results were normalized to *β*-actin and presented as means ± SD (*n* = 3), ∗*p* < 0.05, ∗∗*p* < 0.01, and ∗∗∗*p* < 0.001 VS the indicated groups. (d, e) WB showed the protein level of Traf6, Nfatc1, and Ctsk in OCs after different treatments. Quantitative results were normalized to *β*-actin and presented as means ± SD (*n* = 3). ∗*p* < 0.05, ∗∗*p* < 0.01, and ∗∗∗*p* < 0.001 VS the indicated groups.

To better understand the mechanisms behind the suppression of LPS-stimulated OC differentiation and function that resulted from D7 treatment, we investigated marker gene changes of OCs at both RNA and protein levels. RT-qPCR showed that the expression of OC markers, including differentiation-related (*Traf6*, *Nfatc1*) and function-related (*Ctsk*) markers, were all decreased at mRNA level after D7 treatment in both RANKL-induced and LPS-stimulated conditions (Figures 4(a)–4(c)). These results were further confirmed at the protein level by WB (Figures 4(d) and 4(e)), supporting the suppressive effect of D7 on LPS-stimulated OC differentiation and function at the molecular level.

### 3.5. D7 Promotes OB Differentiation and Alleviates LPS-Induced Osteogenic Damage

**Figure 5:**
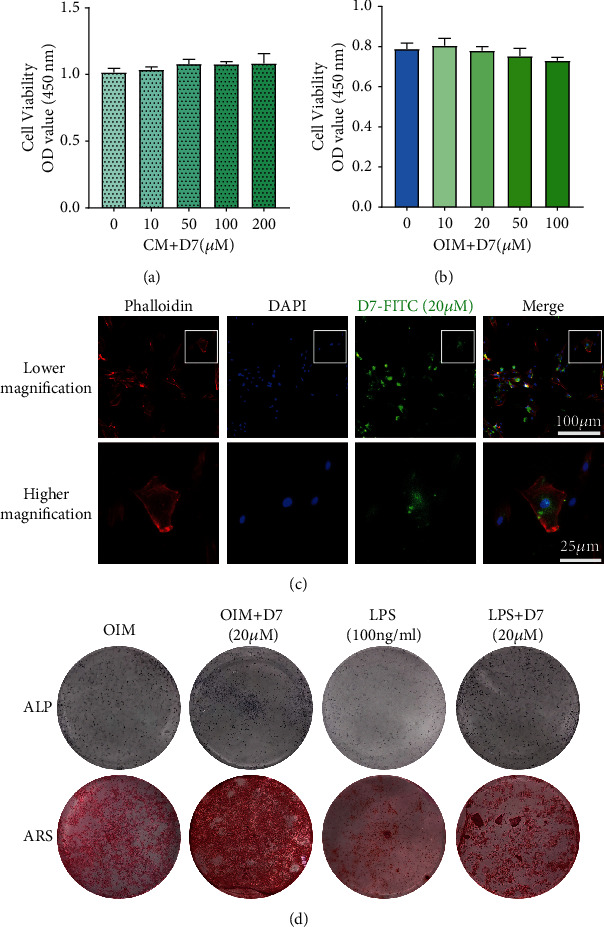
D7 promotes osteoblast (OB) differentiation and alleviates LPS-induced osteogenic damage. (a, b) CCK-8 assay for cell viability test. Calvarial osteoblast precursor cells (OPCs) were stimulated with D7 (0–200 *μ*M) in complete medium (CM) (a) or osteogenic induction medium (OIM) (b) for 48 h (*n* = 3). (c) Fluorescence staining demonstrated the affinity of D7 towards OPCs. The cells induced with FITC-D7 (20 *μ*M) for 48 h were observed under a high-content microscope. The nuclei and cytoskeletons of the cells were counterstained with DAPI and phalloidin-iflour 594, respectively. Scale bar in lower magnification = 100 *μ*m, scale bar in higher magnification = 25 *μ*m. (d) ALP and alizarin red staining of OBs differentiated from OPCs under different stimuli. OPCs were cultured in OIM with or without 100 ng/ml LPS stimulation for 5 days (d, upper panel) or 21 days (d, lower panel), and 20 *μ*M D7 were added to the indicated groups.

Considering the positive role of D7 in suppressing inflammatory osteolysis from the OC aspect, we therefore ask whether D7 could also affect the process of inflammatory osteolysis from the OB aspect. To validate the effect of D7 on OB differentiation and function, cell viability and bioactivity test on OPCs were conducted similar to those on BMMs. Results showed the cell viability of OPCs was not significantly affected when the dosage of D7 was used within 100 *μ*M no matter under OIM condition or not (Figures 5(a) and 5(b)). Similar to BMMs, D7 also demonstrated excellent bioactivity in OPCs and mainly distribute in the cytoplasm (Figure 5(c)), indicating strong bioactivity that may exert during OB differentiation and function.

We then define the effect of D7 on the osteogenic process under LPS conditions. OPCs were cultured and induced by OIM with or without 100 ng/ml LPS for 5 or 21 days, respectively to examine ALP expression and calcium nodules formation. Surprisingly, the application of D7 could promote cellular ALP expression and enhance calcium nodules formation under normal osteogenic induction and displayed rescue effects on ALP activity and nodules formation under LPS-stimulated conditions (Figure 5(d)), indicating a strong therapeutic effect of D7 against LPS-induced osteogenic damage.

### 3.6. D7 Improves the Expression of OB Marker Genes

**Figure 6:**
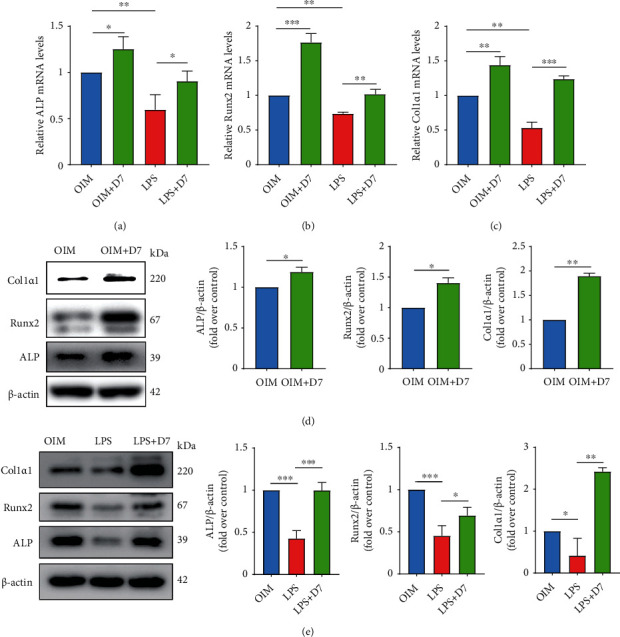
D7 improves the expression of OB marker genes. (a)–(c) RT-qPCR showed the mRNA levels of ALP (a), Runx2 (b), and Col1*α*1 (c) in OPCs. Cells were cultured in OIM with or without 100 ng/ml LPS stimulation for 5 days, and 20 *μ*M D7 was added to the indicated groups. Quantitative results were normalized to *β*-actin and presented as means ± SD (*n* = 3), ∗*p* < 0.05, ∗∗*p* < 0.01, and ∗∗∗*p* < 0.001 VS the indicated groups. (d, e) WB showed the protein level of ALP, Runx2, and Col1a1 in OPCs after different treatments. Quantitative results were normalized to *β*-actin and presented as means ± SD (*n* = 3). ∗*p* < 0.05, ∗∗*p* < 0.01, and ∗∗∗*p* < 0.001 VS the indicated groups.

We next investigated the mechanisms behind the promotive effect on osteogenesis and therapeutic effect against LPS-induced osteogenic damage resulting from D7 application. RT-qPCR showed that the expression of OB markers, including differentiation-related (*ALP*, *Runx2*) and function-related (*Col1α1*) markers, were all increased at mRNA level after D7 application under normal osteogenic induction, and were significantly recovered from LPS-induced reduction (Figures 6(a)–6(c)). These results were further confirmed at the protein level by WB (Figures 6(d) and 6(e)), suggesting a positive impact of D7 on treating inflammatory osteolysis from the OB aspect.

### 3.7. D7 Reduces LPS-Induced ROS Formation in Both Preosteoclast and Preosteoblast

**Figure 7:**
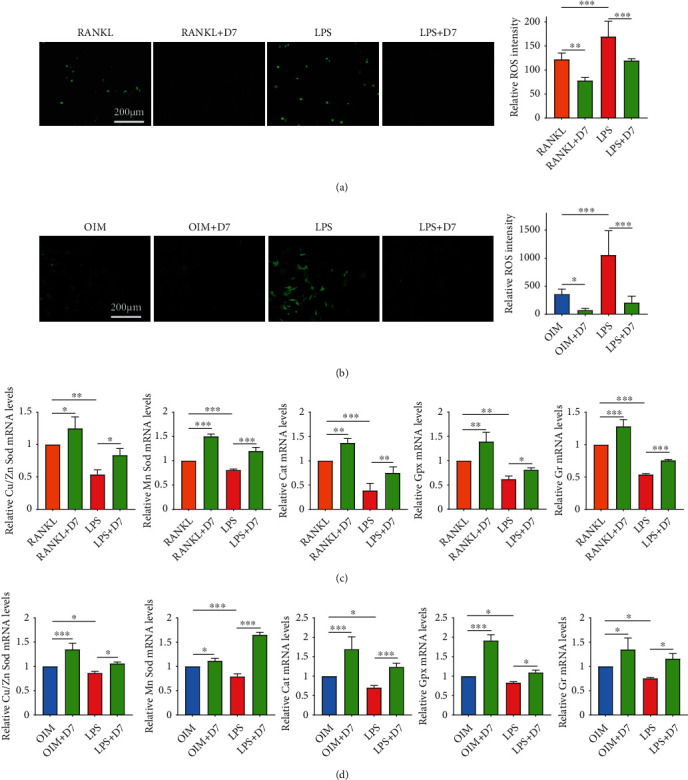
D7 reduces LPS-induced ROS formation in both pre-OC and pre-OB. (a) Fluorescence staining demonstrated the production of ROS in BMMs under different stimuli. BMMs were induced with M-CSF (25 ng/mL) and RANKL (40 ng/mL) for 24 h with or without D7 (20 *μ*M), or stimulated with M-CSF and LPS for 24 h with or without D7 (20 *μ*M). (b) Fluorescence staining verified the level of intracellular ROS in OPCs under different stimuli. Quantitative analysis of ROS fluorescence intensity in (a) and (b) were presented as means ± SD (*n* = 3). ∗*p* < 0.05, ∗∗*p* < 0.01, and ∗∗∗*p* < 0.001 VS indicated groups. Scale bar in (a) and (b) = 200 *μ*m. (c) RT-qPCR showed the mRNA levels of Cu/Zn Sod, Mn Sod, Cat, Gpx, and Gr in BMMs or (d) OPCs under different stimuli as previously described. Quantitative results were normalized to GAPDH and presented as means ± SD (*n* = 3), ∗*p* < 0.05, ∗∗*p* < 0.01, and ∗∗∗*p* < 0.001 VS the indicated groups.

Overproduction of ROS plays an indispensable role in the pathogenesis of inflammatory osteolysis, wherein the elevated formation of ROS leads to hyperactivation of osteoclastogenesis but deterioration of osteogenesis [5, 35]. We, therefore, studied whether the dual effects of D7 on OCs and OBs under LPS-stimulated conditions were due to the unified change of ROS formation. As shown in Figures 7(a) and 7(b), the generation of intracellular ROS in both BMMs and OPCs was robustly increased under LPS stimulation, whereas D7 treatment significantly reduced the ROS formation especially in the scenario of inflammation.

We then identified the responses of the ROS defense system via the evaluation of the antioxidant expression. RT-qPCR showed that LPS reduced the expression of all five antioxidant genes (*Cu/Zn-Sod*, *Mn-Sod*, *Cat*, *Gpx*, and *Gr*), especially in BMMs (Figures 7(c) and 7(d)), indicating the weakening of the antioxidant system on ROS scavenging after LPS stimulation. However, D7 promoted the expression of these antioxidants in normal conditions and restored the expression level under LPS conditions (Figures 7(c) and 7(d)). Taken together, these results exhibited a strong capability of D7 in reducing ROS generation, thereby suppressing LPS-stimulated osteoclastogenesis and alleviating LPS-induced osteogenic damage.

## 4. Discussion

**Figure 8:**
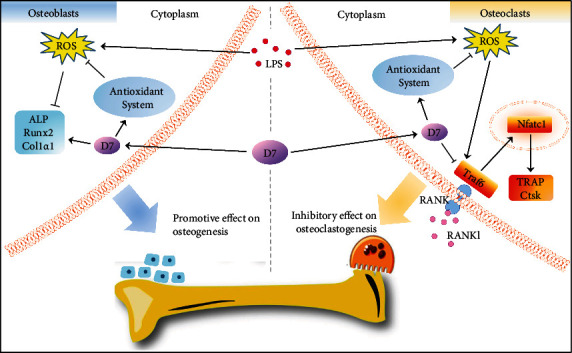
Proposed working mechanisms of cyclic polypeptide D7 on OC and OB during inflammatory osteolysis. Overproduction of ROS in OCs and OBs caused by LPS promotes osteoclastogenesis and inhibits osteogenesis via the effects on cellular marker genes, leading to imbalanced bone remodeling and fragile bones. D7 could restore the bone remodeling balance via the suppression of oxidative stress-induced effects on OCs and OBs, thereby attenuating inflammatory osteolysis. LPS: lipopolysaccharide; ALP: alkaline phosphatase; Runx2: runt-related transcription factor 2; Col1*α*1: type I collagen alpha 1 chain; RANK: TNF receptor superfamily member 11a; RANKL: receptor activator of nuclear factor-*κ*B ligand; Traf6: TNF receptor-associated factor 6; Nfatc1: nuclear factor of activated T-cells 1; Ctsk: cathepsin K; TRAP: tartrate-resistant alkaline phosphatase; ROS: reactive oxygen species.

Characterized by strong specificity, high biological activity, ease of synthesis, and low toxicity, bioactive peptides have gradually accessed to the public visual field due to advantages in medical research [36]. To date, abundance of studies demonstrate the role of polypeptides in treating bone diseases, such as promoting osteogenic differentiation [29] and protecting cells from oxidative stress-induced damage and apoptosis [37]. Compared with linear peptides, cyclic polypeptides provide strong affinity [38], high stability, and better osteointegration due to the cyclic structure [39, 40]. In this study, by using a network pharmacology database and cellular pharmacological validation, we found for the first time that cyclic polypeptide D7 could reduce LPS-induced inflammatory bone loss via the suppression of osteoclastogenesis and promotion on osteogenesis, establishing the potential therapeutic role of D7 in inflammatory bone disease (Figure 8).

Within this study, we verified for the first time that there was a certain connection between the targets of D7 and the pathogenic genes of inflammatory osteolysis through the integrating drug target predictions. From target prediction of D7 and GO/KEGG pathway analysis, our results showed that D7 may regulate OC differentiation and bone resorption via endopeptidase activity, protein binding, and protein kinase binding in the cell surface, plasma membrane, and extracellular region so that to exert its effect on inflammatory response [41, 42]. Since mature OCs are necessary effector cells for inflammation induced-bone loss, OC precursors have become the primary target cells for the prevention and treatment of osteolysis during inflammatory bone diseases [3]. Therefore, a successful validation on the network pharmacology-based analysis of D7 may provide a new option to prevent/treat chronic inflammatory diseases.

We investigated the real effects of D7 on OC differentiation and function under LPS stimulation. LPS-triggered inflammatory status is a well-known model for the study of inflammatory disease progress [34]. LPS promotes osteolysis by stimulating immune cells to release cytokines [43]. Similar to other researches, we also found that the number of OCs increased along with the accumulated concentration of LPS [5]. However, after the pretreatment of D7, the number of OCs decreased notably, regardless of RANKL or LPS-stimulation, as well as the inhibition of the osteolytic function of OCs. Mature OCs possess highly dynamic multi-molecular assemblies composed of F-actin column core (ring-like structure) which is essential for OCs adhesion and motility [30, 44]. Acidic vesicles from the cytoplasm of mature OCs are thereafter secreted into the area sealed by F-actin rings, exerting the degradation on the inorganic bone matrix [31]. We found that both F-actin ring formation and acidified compartment accumulation in LPS-stimulated OCs were reduced after the pretreatment of D7. Key genes that govern OCs differential and function were behind the changes of these cell behaviors. We confirmed that D7 down-regulated the expression of essential genes and proteins for osteoclastogenesis and bone resorption, including Traf6, Nfatc1, and Ctsk [33, 45]. ROS are critical intracellular signaling mediators that connect OC essential genes function, as well as the key regulator of cellular redox state that determines the post-translational modification of protein kinases and phosphatases in OC [46, 47]. In the current study, the level of ROS in BMMs boosted obviously due to LPS stimulation, which is similar to other reports [48]. As the defense system, antioxidant enzymes, could balance the ROS formation, resulting in redox homeostasis within the cell [49, 50]. However, under LPS stimulation, expressions of antioxidant enzymes were suppressed dramatically, whereas we found that D7 could activate the defense system of antioxidants corresponding to the increased generation of ROS during inflammatory osteolysis.

During the process of inflammatory osteolysis, oxidative stress not only excessively activates OCs but also be responsible for OB differentiation failure and cellular apoptosis [6]. The oxidative stress-triggered ROS overproduction could massively deteriorate OB differentiation and function through multiple cellular signaling pathways, and therefore negatively impact bone remodeling [15, 51]. Similar to other studies, we also found a massive generation of intracellular ROS in OPCs under LPS stimulation [6, 52]. However, D7 dramatically reduced LPS-induced ROS in OPCs and increased the expression level of antioxidative-associated substances, which were comparable to the discoveries in BMMs. With this critical finding, it would be no surprise that OB differentiation and function would be benefited from D7 treatment during the process of inflammatory osteolysis [53]. As expected, D7 promoted the expression of Runx2, a key transcription factor for osteogenesis, and increased the level of early and late osteogenic genes, ALP and Col1*α*1, even under LPS conditions [54]. Improvements at the molecular level were also proved at the cellular level, evidenced by the observation of more ALP expression and calcium nodules formation after D7 treatment [55]. Most importantly, we did not notify significant cytotoxicity of D7 on OPCs, even at a comparatively high dosage (200 *μ*M), indicating a good property for further pharmaceutically development. However, there are also certain limitations in our study. Although D7 exhibited a non-negligible therapeutic effect on inflammatory osteolysis, its specific mechanism of action is still unclear, and this study has not further pinpointed the specific target of D7. In future studies, 20 genes obtained from network pharmacology analysis will be biologically verified. Genes that have been preliminary proved to exert a biological regulatory role will be subjected to CRISPER/Cas9 gene editing animal modeling so that to improve the efficiency and success rate of gene-targeting studies [56], thus providing more accurate targets for therapeutic interventions in inflammatory osteolysis.

## 5. Conclusion

Based on comprehensive network pharmacology and pharmacological validation, we demonstrated the therapeutic effect of D7 against LPS-induced inflammatory bone loss via the suppression of oxidative stress and the restoration of the bone remodeling process, from inhibiting osteoclastogenesis to promoting osteogenesis. Our study provides new evidence into the potential therapeutic applications of D7 in the prevention and treatment of chronic inflammatory bone disease.

## Figures and Tables

**Table 1:** Primer sequences used for RT-qPCR.

Gene symbol (GenBank accession no.)	Primers
ALP (NM_007431.3)	F: GCACCTGCCTTACCAACTCT
	R: GTGGAGACGCCCATACCATC
Runx2 (NM_001271631.1)	F: TCAAGGGAATAGAGGGGATGC
	R: GGGAGGACAGAGGGAAACAAC
Col1a1 (NM_007742.4)	F: GACATGTTCAGCTTTGTGGACCTC
	R: GGGACCCTTAGGCCATTGTGTA
Traf6 (NM_001303273.1)	F: AAAGCGAGAGATTCTTTCCCTG
	R: ACTGGGGACAATTCACTAGAGC
Nfatc1 (NM_001164112.1)	F: CCGTTGCTTCCAGAAAATAACA
	R: TGTGGGATGTGAACTCGGAA
Ctsk (NM_007802.4)	F: CTTCCAATACGTGCAGCAGA
	R: TCTTCAGGGCTTTCTCGTTC
Cu/Zn sod (NM_011434.2)	F: AACCAGTTGTGTTGTCAGGAC
	R: CCACCATGTTTCTTAGAGTGAGG
Mn sod (NM_013671.3)	F: TGGACAAACCTGAGCCCTAAG
	R: CCCAAAGTCACGCTTGATAGC
Gpx (NM_001329527.1)	F: AGTCCACCGTGTATGCCTTCT
	R: GAGACGCGACATTCTCAATGA
Gr (NM_010344.4)	F: GCGTGAATGTTGGATGTGTACC
	R: GTTGCATAGCCGTGGATAATTTC
Cat (NM_009804.2)	F: GGAGTCTTCGTCCCGAGTCT
	R: CGGTCTTGTAATGGAACTTGC
GAPDH (NM_001289726.1)	F: ACTTTGTCAAGCTCATTTCC
	R: TGCAGCGAACTTTATTGATG
*β*-Actin (NM_007393.5)	F: CATCCGTAAAGACCTCTATGCCAAC
	R: ATGGAGCCACCGATCCACA

**Table 2:** 20 targets of D7 on inflammatory osteolysis.

Number	Protein name	Gene name
1	Major histocompatibility complex, class I, A	HLA-A
2	Protein tyrosine phosphatase receptor type C	PTPRC
3	Sirtuin 1	SIRT1
4	Calcium sensing receptor	CASR
5	Angiotensin I converting enzyme	ACE
6	Heat shock protein 90 alpha family class A member 1	HSP90AA1
7	Tumor necrosis factor	TNF
8	Fos proto-oncogene, AP-1 transcription factor subunit	FOS
9	C-C motif chemokine receptor 5	CCR5
10	Carbonic anhydrase 2	CA2
11	Spleen associated tyrosine kinase	SYK
12	Platelet derived growth factor receptor beta	PDGFRB
13	Cathepsin D	CTSD
14	Inhibitor of nuclear factor kappa B kinase subunit beta	IKBKB
15	Peptidylprolyl cis/trans isomerase, NIMA-interacting 1	PIN1
16	Farnesyl-diphosphate farnesyltransferase 1	FDFT1
17	ADAM metallopeptidase domain 8	ADAM8
18	ALK receptor tyrosine kinase	ALK
19	Matrix metallopeptidase 10	MMP10
20	Matrix metallopeptidase 12	MMP12

## Data Availability

The data used in the study to support main findings will be available from the corresponding author upon request.

## Supplementary Materials

Supplementary 1 Supplement Table 1: 100 putative targets were retrieved from the Swiss Target Prediction database based on the structure of D7.

Supplementary 2 Supplement Table 2: 1219 inflammatory osteolysis-related human genes were collected from the GeneCards database using “Inflammatory osteolysis” as keywords.
