# Supplementary material for: Network Pharmacology Deciphers the Action of Bioactive Polypeptide in Attenuating Inflammatory Osteolysis via the Suppression of Oxidative Stress and Restoration of Bone Remodeling Balance
Source: Oxid Med Cell Longev. 2022 Apr 14;2022:4913534. doi: 10.1155/2022/4913534 (PMC9107052; doi:10.1155/2022/4913534)
Supplement: Supplementary 1 — Supplement Table 1: 100 putative targets were retrieved from the Swiss Target Prediction database based on the structure of D7. [file 4913534.f1.pdf]

**Supplement Table 1. 100 putative targets of D7.**

| Number | Protein name | Gene name |
|---|---|---|
| 1 | Integrin alpha-3 | ITGA3 |
| 2 | Integrin alpha-4/beta-1 | ITGB1 ITGA4 |
| 3 | Integrin alpha-4/beta-7 | ITGB7 ITGA4 |
| 4 | Integrin alpha-IIb/beta-3 | ITGA2B ITGB3 |
| 5 | Integrin alpha-V/beta-3 | ITGAV ITGB3 |
| 6 | Integrin alpha-5/beta-1 | ITGB1 ITGA5 |
| 7 | SPRY domain-containing SOCS box protein 2 | SPSB2 |
| 8 | Oxytocin receptor (by homology) | OXTR |
| 9 | Beta-secretase 1 | BACE1 |
| 10 | HLA class I histocompatibility antigen A-3 | HLA-A |
| 11 | Mu opioid receptor (by homology) | OPRM1 |
| 12 | Adenosine A2a receptor | ADORA2A |
| 13 | Integrin alpha-4 | ITGA4 |
| 14 | Delta opioid receptor (by homology) | OPRD1 |
| 15 | Vasopressin V1b receptor | AVPR1B |
| 16 | DNA (cytosine-5)-methyltransferase 1 | DNMT1 |
| 17 | DNA (cytosine-5)-methyltransferase 3B | DNMT3B |
| 18 | C3a anaphylatoxin chemotactic receptor | C3AR1 |
| 19 | Leukocyte common antigen | PTPRC |
| 20 | Aminopeptidase A | ENPEP |
| 21 | Inhibitor of apoptosis protein 3 | XIAP |
| 22 | Thromboxane-A synthase | TBXAS1 |
| 23 | Cholecystokinin A receptor | CCKAR |
| 24 | Baculoviral IAP repeat-containing protein 3 | BIRC3 |
| 25 | CD22 | CD22 |
| 26 | Disks large homolog 4 | DLG4 |
| 27 | Protein-tyrosine phosphatase 1B | PTPN1 |
| 28 | T-cell protein-tyrosine phosphatase | PTPN2 |
| 29 | NAD-dependent deacetylase sirtuin 1 | SIRT1 |
| 30 | Melanocortin receptor 4 | MC4R |
| 31 | Insulin-degrading enzyme | IDE |
| 32 | Purinergic receptor P2Y12 | P2RY12 |
| 33 | Growth factor receptor-bound protein 2 | GRB2 |
| 34 | Calcium sensing receptor | CASR |
| 35 | Vasopressin V2 receptor (by homology) | AVPR2 |
| 36 | Vasopressin V1a receptor (by homology) | AVPR1A |
| 37 | Scavenger receptor class B member 1 | SCARB1 |
| 38 | Angiotensin-converting enzyme | ACE |
| 39 | Histone-lysine N-methyltransferase, H3 lysine-79 specific | DOT1L |
| 40 | Casein kinase II alpha | CSNK2A1 |
| 41 | Heat shock protein HSP 90-alpha | HSP90AA1 |
| 42 | Casein kinase II alpha (prime) | CSNK2A2 |

| | | |
|---|---|---|
| 43 | Serine/threonine protein phosphatase 2A, catalytic subunit, alpha isoform | PPP2CA |
| 44 | P-selectin | SELP |
| 45 | Serine/threonine-protein phosphatase | PPP5C |
| 46 | TNF-alpha | TNF |
| 47 | Serine/threonine protein phosphatase PP1-alpha catalytic subunit | PPP1CA |
| 48 | Transcription factor AP1 | FOS |
| 49 | C-C chemokine receptor type 5 | CCR5 |
| 50 | Tyrosine-protein kinase LCK | LCK |
| 51 | Ileal bile acid transporter | SLC10A2 |
| 52 | Carbonic anhydrase II | CA2 |
| 53 | Beta-3 adrenergic receptor | ADRB3 |
| 54 | Carbonic anhydrase I | CA1 |
| 55 | Adenosine A1 receptor | ADORA1 |
| 56 | Adenosine A3 receptor | ADORA3 |
| 57 | Tyrosine-protein kinase SYK | SYK |
| 58 | Myelin-associated glycoprotein | MAG |
| 59 | Platelet-derived growth factor receptor beta | PDGFRB |
| 60 | Thymidylate synthase (by homology) | TYMS |
| 61 | Protein phosphatase 2C beta | PPM1B |
| 62 | Serine/threonine protein phosphatase PP1-gamma catalytic subunit | PPP1CC |
| 63 | Serine/threonine protein phosphatase 2A, 56 kDa regulatory subunit, alpha isoform | PPP2R5A |
| 64 | Lysosomal protective protein | CTSA |
| 65 | Cathepsin D | CTSD |
| 66 | Inhibitor of nuclear factor kappa B kinase beta subunit | IKBKB |
| 67 | EZH2/SUZ12/EED/RBBP7/RBBP4 | EZH2 |
| 68 | Peptidyl-prolyl cis-trans isomerase NIMA-interacting 1 | PIN1 |
| 69 | Tissue-type plasminogen activator | PLAT |
| 70 | Coagulation factor XI | F11 |
| 71 | Coagulation factor VII | F7 |
| 72 | Peroxisome proliferator-activated receptor gamma | PPARG |
| 73 | Squalene synthetase (by homology) | FDFT1 |
| 74 | GABA transporter 1 (by homology) | SLC6A1 |
| 75 | HERG | KCNH2 |
| 76 | Disintegrin and metalloproteinase domain-containing protein 8 | ADAM8 |
| 77 | Glutamate receptor ionotropic kainate 1 | GRIK1 |
| 78 | Glutamate receptor ionotropic kainate 2 | GRIK2 |
| 79 | Glutamate receptor ionotropic kainate 3 | GRIK3 |
| 80 | ALK tyrosine kinase receptor | ALK |
| 81 | Serotonin 2b (5-HT2b) receptor | HTR2B |
| 82 | Alpha-2a adrenergic receptor | ADRA2A |

| | | |
|---|---|---|
| 83 | Adrenergic receptor alpha-2 | ADRA2C |
| 84 | Alpha-2b adrenergic receptor | ADRA2B |
| 85 | Dopamine D1 receptor | DRD1 |
| 86 | Dopamine D2 receptor | DRD2 |
| 87 | Alpha-1d adrenergic receptor | ADRA1D |
| 88 | Serotonin 2a (5-HT2a) receptor | HTR2A |
| 89 | Serotonin 2c (5-HT2c) receptor | HTR2C |
| 90 | Alpha-1a adrenergic receptor (by homology) | ADRA1A |
| 91 | Dopamine D3 receptor | DRD3 |
| 92 | Peroxisome proliferator-activated receptor alpha | PPARA |
| 93 | Cytochrome P450 2D6 | CYP2D6 |
| 94 | Serotonin 6 (5-HT6) receptor | HTR6 |
| 95 | Sodium/glucose cotransporter 2 | SLC5A2 |
| 96 | Matrix metalloproteinase 10 | MMP10 |
| 97 | Matrix metalloproteinase 12 | MMP12 |
| 98 | Serotonin 1b (5-HT1b) receptor (by homology) | HTR1B |
| 99 | Kallikrein 1 | KLK1 |
| 100 | Alpha-1b adrenergic receptor | ADRA1B |
