# Supplementary material for: Network Pharmacology Deciphers the Action of Bioactive Polypeptide in Attenuating Inflammatory Osteolysis via the Suppression of Oxidative Stress and Restoration of Bone Remodeling Balance
Source: Oxid Med Cell Longev. 2022 Apr 14;2022:4913534. doi: 10.1155/2022/4913534 (PMC9107052; doi:10.1155/2022/4913534)
Supplement: Supplementary 2 — Supplement Table 2: 1219 inflammatory osteolysis-related human genes were collected from the GeneCards database using “Inflammatory osteolysis” as keywords. [file 4913534.f2.pdf]

Supplement Table 2. 1219 human genes of inflammatory osteolysis-related.

Number	Protein name	Gene name
1	Matrix Metallopeptidase 2	MMP2
2	Nucleotide Binding Oligomerization Domain Containing 2	NOD2
3	Interleukin 10	IL10
4	Interleukin 6	IL6
5	MAF BZIP Transcription Factor B	MAFB
6	Tumor Necrosis Factor	TNF
7	TNF Receptor Superfamily Member 11a	TNFRSF11A
8	Transforming Growth Factor Beta 1	TGFB1
9	ADAM Metallopeptidase Domain 17	ADAM17
10	Lysophosphatidylcholine Acyltransferase 2	LPCAT2
11	Interleukin 1 Beta	IL1B
12	Matrix Metallopeptidase 14	MMP14
13	C-X-C Motif Chemokine Ligand 8	CXCL8
14	Receptor Interacting Serine/Threonine Kinase 1	RIPK1
15	NLR Family Pyrin Domain Containing 3	NLRP3
16	Interleukin 23 Receptor	IL23R
17	Toll Like Receptor 4	TLR4
18	Interferon Gamma	IFNG
19	Major Histocompatibility Complex, Class II, DR Beta 1	HLA-DRB1
20	C-Reactive Protein	CRP
21	Prostaglandin-Endoperoxide Synthase 2	PTGS2
22	Epidermal Growth Factor Receptor	EGFR
23	C-C Motif Chemokine Ligand 2	CCL2
24	Interleukin 1 Receptor Antagonist	IL1RN
25	C-C Motif Chemokine Ligand 3	CCL3
26	Interleukin 1 Alpha	IL1A
27	Interferon Regulatory Factor 5	IRF5
28	TNF Superfamily Member 11	TNFSF11
29	Interleukin 4	IL4
30	Interleukin 17A	IL17A
31	TNF Receptor Superfamily Member 1A	TNFRSF1A
32	ATP Binding Cassette Subfamily B Member 1	ABCB1
33	Interleukin 13	IL13
34	BAF Nuclear Assembly Factor 1	BANF1
35	MEFV Innate Immuity Regulator, Pyrin	MEFV
36	Major Histocompatibility Complex, Class I, B	HLA-B
37	Interleukin 2	IL2
38	C-C Motif Chemokine Receptor 6	CCR6
39	Proline-Serine-Threonine Phosphatase Interacting Protein 1	PSTPIP1
40	C-C Motif Chemokine Ligand 4	CCL4
41	Notch Receptor 2	NOTCH2
42	Toll Like Receptor 2	TLR2

43	TNF Receptor Superfamily Member 11b	TNFRSF11B
44	Tumor Protein P53	TP53
45	Intercellular Adhesion Molecule 1	ICAM1
46	Cytotoxic T-Lymphocyte Associated Protein 4	CTLA4
47	Interleukin 2 Receptor Subunit Alpha	IL2RA
48	Protein Tyrosine Phosphatase Non-Receptor Type 22	PTPN22
49	C-C Motif Chemokine Receptor 1	CCR1
50	Interleukin 12B	IL12B
51	Mitogen-Activated Protein Kinase 14	MAPK14
52	Prostaglandin-Endoperoxide Synthase 1	PTGS1
53	C-C Motif Chemokine Ligand 5	CCL5
54	Signal Transducer And Activator Of Transcription 1	STAT1
55	Lamin A/C	LMNA
56	Vascular Endothelial Growth Factor A	VEGFA
57	Interferon Induced With Helicase C Domain 1	IFIH1
58	Lymphotoxin Alpha	LTA
59	Matrix Metallopeptidase 9	MMP9
60	Platelet Derived Growth Factor Receptor Alpha	PDGFRA
61	Macrophage Migration Inhibitory Factor	MIF
62	Arachidonate 5-Lipoxygenase	ALOX5
63	C-C Motif Chemokine Receptor 5	CCR5
64	Interleukin 1 Receptor Type 1	IL1R1
65	Spleen Associated Tyrosine Kinase	SYK
66	C-C Motif Chemokine Ligand 11	CCL11
67	Secreted Phosphoprotein 1	SPP1
68	Sequestosome 1	SQSTM1
69	Interleukin 5	IL5
70	Signal Transducer And Activator Of Transcription 3	STAT3
71	C-C Motif Chemokine Ligand 20	CCL20
72	Interleukin 18	IL18
73	ALK Receptor Tyrosine Kinase	ALK
74	Matrix Metallopeptidase 1	MMP1
75	Nuclear Factor Kappa B Subunit 1	NFKB1
76	Elastase, Neutrophil Expressed	ELANE
77	Zinc Metallopeptidase STE24	ZMPSTE24
78	Interleukin 6 Receptor	IL6R
79	NFKB Inhibitor Alpha	NFKBIA
80	C-X-C Motif Chemokine Ligand 10	CXCL10
81	Colony Stimulating Factor 1	CSF1
82	Platelet Derived Growth Factor Receptor Beta	PDGFRB
83	Selectin E	SELE
84	Peripheral Myelin Protein 22	PMP22
85	TNF Receptor Superfamily Member 1B	TNFRSF1B
86	Albumin	ALB

87	C-X-C Motif Chemokine Ligand 2	CXCL2
88	Adiponectin, C1Q And Collagen Domain Containing	ADIPOQ
89	Signal Transducer And Activator Of Transcription 4	STAT4
90	Proteinase 3	PRTN3
91	Fas Cell Surface Death Receptor	FAS
92	Nitric Oxide Synthase 2	NOS2
93	Inhibitor Of Nuclear Factor Kappa B Kinase Regulatory Subunit Gamma	IKBKG
94	Colony Stimulating Factor 2	CSF2
95	MicroRNA 146a	MIR146A
96	Vascular Cell Adhesion Molecule 1	VCAM1
97	Major Histocompatibility Complex, Class I, C	HLA-C
98	CD40 Molecule	CD40
99	Leptin	LEP
100	C-X-C Motif Chemokine Receptor 4	CXCR4
101	Inhibitor Of Nuclear Factor Kappa B Kinase Subunit Beta	IKBKB
102	CD40 Ligand	CD40LG
103	Janus Kinase 2	JAK2
104	MYD88 Innate Immune Signal Transduction Adaptor	MYD88
105	Interleukin 15	IL15
106	Caspase 1	CASP1
107	Glucosamine (UDP-N-Acetyl)-2-Epimerase/N-Acetylmannosamine Kinase	GNE
108	Calcitonin Related Polypeptide Alpha	CALCA
109	Matrix Metallopeptidase 3	MMP3
110	Major Histocompatibility Complex, Class II, DQ Alpha 1	HLA-DQA1
111	C-X-C Motif Chemokine Ligand 9	CXCL9
112	Vitamin D Receptor	VDR
113	15-Hydroxyprostaglandin Dehydrogenase	HPGD
114	APC Regulator Of WNT Signaling Pathway	APC
115	Endoplasmic Reticulum Aminopeptidase 1	ERAP1
116	Interleukin 2 Receptor Subunit Beta	IL2RB
117	Notch Receptor 3	NOTCH3
118	Interleukin 22	IL22
119	Coagulation Factor V	F5
120	C-X-C Motif Chemokine Ligand 1	CXCL1
121	MicroRNA 155	MIR155
122	Interleukin 36 Receptor Antagonist	IL36RN
123	Catenin Beta 1	CTNNB1
124	Toll Like Receptor 3	TLR3
125	Complement C4A (Rodgers Blood Group)	C4A
126	Major Histocompatibility Complex, Class II, DP Beta 1	HLA-DPB1
127	Adenosine Deaminase	ADA
128	TIMP Metallopeptidase Inhibitor 1	TIMP1

129	Jun Proto-Oncogene, AP-1 Transcription Factor Subunit	JUN
130	S100 Calcium Binding Protein A12	S100A12
131	Interferon Beta 1	IFNB1
132	CD163 Molecule	CD163
133	Caspase Recruitment Domain Family Member 14	CARD14
134	Interleukin 1 Receptor Accessory Protein Like 2	IL1RAPL2
135	Interleukin 11	IL11
136	C-X-C Motif Chemokine Receptor 3	CXCR3
137	C-X-C Motif Chemokine Ligand 12	CXCL12
138	Bone Gamma-Carboxyglutamate Protein	BGLAP
139	CD4 Molecule	CD4
140	Platelet And Endothelial Cell Adhesion Molecule 1	PECAM1
141	Cathepsin K	CTSK
142	Colony Stimulating Factor 3	CSF3
143	AKT Serine/Threonine Kinase 1	AKT1
144	Inducible T Cell Costimulator Ligand	ICOSLG
145	Insulin	INS
146	Hypoxia Inducible Factor 1 Subunit Alpha	HIF1A
147	Interleukin 3	IL3
148	Component Of Inhibitor Of Nuclear Factor Kappa B Kinase Complex	CHUK
149	MicroRNA 21	MIR21
150	Matrix Metallopeptidase 13	MMP13
151	CD36 Molecule	CD36
152	Cellular Communication Network Factor 2	CCN2
153	Fc Fragment Of IgG Receptor IIa	FCGR2A
154	Lipin 2	LPIN2
155	Serpin Family E Member 1	SERPINE1
156	Apolipoprotein E	APOE
157	Major Histocompatibility Complex, Class I, A	HLA-A
158	Mitogen-Activated Protein Kinase 1	MAPK1
159	C-Type Lectin Domain Containing 7A	CLEC7A
160	KRAS Proto-Oncogene, GTPase	KRAS
161	Membrane Bound Transcription Factor Peptidase, Site 2	MBTPS2
162	Zeta Chain Of T Cell Receptor Associated Protein Kinase 70	ZAP70
163	Caspase 8	CASP8
164	Acid Phosphatase 5, Tartrate Resistant	ACP5
165	Interleukin 27	IL27
166	SH3 And PX Domains 2B	SH3PXD2B
167	Interleukin 7	IL7
168	Cathepsin C	CTSC
169	Interleukin 4 Receptor	IL4R
170	Selectin L	SELL
171	Interleukin 23 Subunit Alpha	IL23A

172	Complement C3d Receptor 2	CR2
173	Phospholipase A2 Group IVA	PLA2G4A
174	TRAF3 Interacting Protein 2	TRAF3IP2
175	Estrogen Receptor 1	ESR1
176	C-C Motif Chemokine Receptor 3	CCR3
177	C-C Motif Chemokine Ligand 18	CCL18
178	Interleukin 6 Cytokine Family Signal Transducer	IL6ST
179	Caspase Recruitment Domain Family Member 9	CARD9
180	Coagulation Factor II, Thrombin	F2
181	Glucosylceramidase Beta	GBA
182	MYC Proto-Oncogene, BHLH Transcription Factor	MYC
183	KIT Proto-Oncogene, Receptor Tyrosine Kinase	KIT
184	Hepatocyte Growth Factor	HGF
185	Caspase 3	CASP3
186	Fc Fragment Of IgG Receptor IIIa	FCGR3A
187	Myoglobin	MB
188	Fos Proto-Oncogene, AP-1 Transcription Factor Subunit	FOS
189	Complement C1s	C1S
190	Suppressor Of Cytokine Signaling 1	SOCS1
191	Fc Fragment Of IgG Receptor IIIb	FCGR3B
192	Collagen Type III Alpha 1 Chain	COL3A1
193	TNF Receptor Associated Factor 6	TRAF6
194	Cyclin Dependent Kinase Inhibitor 1A	CDKN1A
195	Heat Shock Protein Family D (Hsp60) Member 1	HSPD1
196	Sodium Voltage-Gated Channel Alpha Subunit 9	SCN9A
197	Discoidin Domain Receptor Tyrosine Kinase 2	DDR2
198	Protein Tyrosine Phosphatase Receptor Type C	PTPRC
199	Methylenetetrahydrofolate Reductase	MTHFR
200	Integrin Subunit Alpha L	ITGAL
201	Suppressor Of Cytokine Signaling 3	SOCS3
202	CAMP Responsive Element Binding Protein 1	CREB1
203	Fibroblast Growth Factor 2	FGF2
204	Angiotensin I Converting Enzyme	ACE
205	TNFAIP3 Interacting Protein 1	TNIP1
206	Secretory Leukocyte Peptidase Inhibitor	SLPI
207	CD80 Molecule	CD80
208	Capping Protein Regulator And Myosin 1 Linker 2	CARMIL2
209	Cathelicidin Antimicrobial Peptide	CAMP
210	Mannose Binding Lectin 2	MBL2
211	MicroRNA 223	MIR223
212	RB Transcriptional Corepressor 1	RB1
213	Gap Junction Protein Beta 2	GJB2
214	Cyclin D1	CCND1
215	Serpin Family A Member 3	SERPINA3

216	Transforming Growth Factor Beta Receptor 2	TGFBR2
217	Insulin Like Growth Factor 1	IGF1
218	Colony Stimulating Factor 1 Receptor	CSF1R
219	Collagen Type I Alpha 1 Chain	COL1A1
220	CD8a Molecule	CD8A
221	Cytochrome C Oxidase Subunit 5A	COX5A
222	PYD And CARD Domain Containing	PYCARD
223	CD79a Molecule	CD79A
224	Interleukin 33	IL33
225	Advanced Glycosylation End-Product Specific Receptor	AGER
226	Thrombospondin 1	THBS1
227	Epidermal Growth Factor	EGF
228	Solute Carrier Organic Anion Transporter Family Member 2A1	SLCO2A1
229	GNAS Complex Locus	GNAS
230	IL2 Inducible T Cell Kinase	ITK
231	Interferon Alpha 1	IFNA1
232	BCL2 Apoptosis Regulator	BCL2
233	Interleukin 17 Receptor A	IL17RA
234	Phosphatidylinositol-4,5-Bisphosphate 3-Kinase Catalytic Subunit Alpha	PIK3CA
235	Prostaglandin E Receptor 4	PTGER4
236	Ghrelin And Obestatin Prepropeptide	GHRL
237	Kininogen 1	KNG1
238	Interleukin 9	IL9
239	Transient Receptor Potential Cation Channel Subfamily V Member 1	TRPV1
240	Notch Receptor 1	NOTCH1
241	C-C Motif Chemokine Ligand 17	CCL17
242	Bruton Tyrosine Kinase	BTK
243	Prostaglandin E Synthase	PTGES
244	Cathepsin G	CTSG
245	Bone Morphogenetic Protein 6	BMP6
246	Complement C4B (Chido Blood Group)	C4B
247	Parathyroid Hormone Like Hormone	PTHLH
248	CD86 Molecule	CD86
249	CD34 Molecule	CD34
250	LIF Interleukin 6 Family Cytokine	LIF
251	DExD/H-Box Helicase 58	DDX58
252	Erythropoietin	EPO
253	Actin Alpha Cardiac Muscle 1	ACTC1
254	Neurofibromin 1	NF1
255	Ceruloplasmin	CP
256	Fibrillin 1	FBN1
257	Valosin Containing Protein	VCP

258	CCAAT Enhancer Binding Protein Beta	CEBPB
259	Erb-B2 Receptor Tyrosine Kinase 2	ERBB2
260	Signal Transducer And Activator Of Transcription 6	STAT6
261	MicroRNA 126	MIR126
262	Reticulophagy Regulator 1	RETREG1
263	Heat Shock Protein Family A (Hsp70) Member 4	HSPA4
264	Coagulation Factor II Thrombin Receptor	F2R
265	Solute Carrier Family 29 Member 3	SLC29A3
266	RUNX Family Transcription Factor 2	RUNX2
267	Phosphatase And Tensin Homolog	PTEN
268	Cathepsin B	CTSB
269	SAM And HD Domain Containing Deoxynucleoside Triphosphate Triphosphohydrolase 1	SAMHD1
270	Lymphatic Vessel Endothelial Hyaluronan Receptor 1	LYVE1
271	Adaptor Related Protein Complex 1 Subunit Sigma 3	AP1S3
272	Hydroxysteroid 11-Beta Dehydrogenase 1	HSD11B1
273	Vasoactive Intestinal Peptide	VIP
274	Beta-2-Microglobulin	B2M
275	Sirtuin 1	SIRT1
276	Nerve Growth Factor	NGF
277	Caveolin 1	CAV1
278	MDM2 Proto-Oncogene	MDM2
279	Transforming Growth Factor Beta Receptor 1	TGFBR1
280	Cyclin Dependent Kinase Inhibitor 1B	CDKN1B
281	CD68 Molecule	CD68
282	Vimentin	VIM
283	Tachykinin Precursor 1	TAC1
284	CD44 Molecule (Indian Blood Group)	CD44
285	Glucuronidase Beta	GUSB
286	Parathyroid Hormone	PTH
287	Checkpoint Kinase 2	CHEK2
288	Oncostatin M	OSM
289	Lamin B Receptor	LBR
290	Granzyme B	GZMB
291	GATA Binding Protein 1	GATA1
292	CD69 Molecule	CD69
293	TIMP Metallopeptidase Inhibitor 2	TIMP2
294	Brain Derived Neurotrophic Factor	BDNF
295	Proteoglycan 4	PRG4
296	Metastasis Associated Lung Adenocarcinoma Transcript 1	MALAT1
297	Recombination Activating 1	RAG1
298	Ret Proto-Oncogene	RET
299	Interferon Alpha 2	IFNA2
300	Proopiomelanocortin	POMC

301	Uroporphyrinogen III Synthase	UROS
302	MET Proto-Oncogene, Receptor Tyrosine Kinase	MET
303	Fibroblast Growth Factor Receptor 1	FGFR1
304	MHC Class I Polypeptide-Related Sequence A	MICA
305	Recombination Activating 2	RAG2
306	Elastin	ELN
307	ATM Serine/Threonine Kinase	ATM
308	C-C Motif Chemokine Ligand 27	CCL27
309	Proteasome 20S Subunit Beta 9	PSMB9
310	BCL2 Like 1	BCL2L1
311	SRC Proto-Oncogene, Non-Receptor Tyrosine Kinase	SRC
312	ANTXR Cell Adhesion Molecule 2	ANTXR2
313	Phosphatidylinositol-4-Phosphate 3-Kinase Catalytic Subunit Type 2 Alpha	PIK3C2A
314	ATPase Copper Transporting Alpha	ATP7A
315	REL Proto-Oncogene, NF-KB Subunit	REL
316	Secreted Protein Acidic And Cysteine Rich	SPARC
317	Keratin 14	KRT14
318	C-C Motif Chemokine Receptor 4	CCR4
319	X-Box Binding Protein 1	XBP1
320	Heterogeneous Nuclear Ribonucleoprotein A1	HNRNPA1
321	Vascular Endothelial Growth Factor C	VEGFC
322	Endothelin 1	EDN1
323	Purinergic Receptor P2X 7	P2RX7
324	Leucine Rich Repeat Kinase 2	LRRK2
325	Matrix Metallopeptidase 7	MMP7
326	Chromogranin A	CHGA
327	Nicotinamide Phosphoribosyltransferase	NAMPT
328	Fibroblast Growth Factor 7	FGF7
329	Rac Family Small GTPase 1	RAC1
330	Serpin Family A Member 1	SERPINA1
331	Amphiregulin	AREG
332	Fms Related Receptor Tyrosine Kinase 4	FLT4
333	Cyclin Dependent Kinase 4	CDK4
334	Interleukin 1 Receptor Like 2	IL1RL2
335	Growth Factor Independent 1 Transcriptional Repressor	GFI1
336	SMAD Family Member 3	SMAD3
337	Hemopexin	HPX
338	SMAD Family Member 7	SMAD7
339	Parathyroid Hormone 1 Receptor	PTH1R
340	Transferrin Receptor	TFRC
341	Kelch Domain Containing 8B	KLHDC8B
342	C-C Motif Chemokine Ligand 15	CCL15
343	Keratin 10	KRT10

344	Fibroblast Growth Factor 1	FGF1
345	Superoxide Dismutase 2	SOD2
346	TEK Receptor Tyrosine Kinase	TEK
347	Gap Junction Protein Beta 6	GJB6
348	Plasminogen Activator, Urokinase	PLAU
349	H2A Clustered Histone 18	H2AC18
350	Secretin	SCT
351	Toll Like Receptor 8	TLR8
352	Desmin	DES
353	Procollagen-Lysine,2-Oxoglutarate 5-Dioxygenase 1	PLOD1
354	NADH:Ubiquinone Oxidoreductase Complex Assembly Factor 3	NDUFAF3
355	Vasodilator Stimulated Phosphoprotein	VASP
356	Cannabinoid Receptor 2	CNR2
357	Cyclin Dependent Kinase Inhibitor 2A	CDKN2A
358	TNF Receptor Superfamily Member 10a	TNFRSF10A
359	RAB11 Binding And LisH Domain, Coiled-Coil And HEAT Repeat Containing	RELCH
360	TNF Superfamily Member 10	TNFSF10
361	C-X-C Motif Chemokine Ligand 13	CXCL13
362	CD209 Molecule	CD209
363	MicroRNA 17	MIR17
364	SH2 Domain Containing 1A	SH2D1A
365	Intelectin 1	ITLN1
366	Matrix Metallopeptidase 8	MMP8
367	Synuclein Alpha	SNCA
368	Cytochrome C, Somatic	CYCS
369	Galectin 3	LGALS3
370	TNF Superfamily Member 13b	TNFSF13B
371	Cell Adhesion Associated, Oncogene Regulated	CDON
372	MicroRNA 140	MIR140
373	KIT Ligand	KITLG
374	Transferrin	TF
375	Lysine Demethylase 4C	KDM4C
376	Menin 1	MEN1
377	Lamin B1	LMNB1
378	ETS Proto-Oncogene 1, Transcription Factor	ETS1
379	Isocitrate Dehydrogenase (NADP(+)) 1	IDH1
380	Prostaglandin E Receptor 2	PTGER2
381	Toll Like Receptor 7	TLR7
382	Mitogen-Activated Protein Kinase 3	MAPK3
383	Annexin A5	ANXA5
384	TNF Receptor Associated Factor 2	TRAF2
385	Marker Of Proliferation Ki-67	MKI67
386	Angiopoietin 2	ANGPT2

387	Plasminogen Activator, Urokinase Receptor	PLAUR
388	TNF Superfamily Member 12	TNFSF12
389	ATP Binding Cassette Subfamily C Member 1	ABCC1
390	MicroRNA 22	MIR22
391	Alpha Glucosidase	GAA
392	Recombination Signal Binding Protein For Immunoglobulin Kappa J Region	RBPJ
393	Collagen Type II Alpha 1 Chain	COL2A1
394	Podoplanin	PDPN
395	Glucagon	GCG
396	Fibroblast Growth Factor Receptor 3	FGFR3
397	C-C Motif Chemokine Receptor 7	CCR7
398	Heterogeneous Nuclear Ribonucleoprotein A2/B1	HNRNPA2B1
399	Heat Shock Protein 90 Alpha Family Class A Member 1	HSP90AA1
400	Deoxyhypusine Hydroxylase	DOHH
401	Adenylate Cyclase 10	ADCY10
402	Ferredoxin 1	FDX1
403	Interleukin 12 Receptor Subunit Beta 1	IL12RB1
404	Interferon Gamma Receptor 1	IFNGR1
405	Gap Junction Protein Alpha 1	GJA1
406	ADAM Metallopeptidase With Thrombospondin Type 1 Motif 4	ADAMTS4
407	Apolipoprotein H	APOH
408	Mitogen-Activated Protein Kinase Kinase Kinase 7	MAP3K7
409	Tet Methylcytosine Dioxygenase 2	TET2
410	Cyclin Dependent Kinase Inhibitor 2B	CDKN2B
411	TNF Receptor Superfamily Member 6b	TNFRSF6B
412	Semaphorin 4D	SEMA4D
413	Gamma-Glutamyltransferase 1	GGT1
414	Nuclear Factor Kappa B Subunit 2	NFKB2
415	Signal Transducer And Activator Of Transcription 5B	STAT5B
416	Baculoviral IAP Repeat Containing 2	BIRC2
417	Transient Receptor Potential Cation Channel Subfamily V Member 4	TRPV4
418	Signal Transducer And Activator Of Transcription 5A	STAT5A
419	Homeostatic Iron Regulator	HFE
420	AT-Rich Interaction Domain 1B	ARID1B
421	Heat Shock Protein Family A (Hsp70) Member 1A	HSPA1A
422	Glutathione S-Transferase Mu 1	GSTM1
423	Integrin Subunit Beta 1	ITGB1
424	Uroporphyrinogen Decarboxylase	UROD
425	Adaptor Related Protein Complex 4 Subunit Mu 1	AP4M1
426	Small EDRK-Rich Factor 1A	SERF1A
427	Small EDRK-Rich Factor 1B	SERF1B
428	Zinc Finger E-Box Binding Homeobox 1	ZEB1

429	CASP8 And FADD Like Apoptosis Regulator	CFLAR
430	Jagged Canonical Notch Ligand 1	JAG1
431	Insulin Like Growth Factor 1 Receptor	IGF1R
432	Aggrecan	ACAN
433	Heparanase	HPSE
434	WNK Lysine Deficient Protein Kinase 1	WNK1
435	TNF Receptor Superfamily Member 8	TNFRSF8
436	Bone Morphogenetic Protein 2	BMP2
437	Phosphoinositide-3-Kinase Regulatory Subunit 1	PIK3R1
438	Mannose Receptor C-Type 1	MRC1
439	Retinol Binding Protein 4	RBP4
440	Renin	REN
441	B-Raf Proto-Oncogene, Serine/Threonine Kinase	BRAF
442	Scavenger Receptor Class B Member 2	SCARB2
443	BCL2 Associated X, Apoptosis Regulator	BAX
444	Isocitrate Dehydrogenase (NADP(+)) 2	IDH2
445	Insulin Like Growth Factor Binding Protein 3	IGFBP3
446	Proteasome Maturation Protein	POMP
447	Ras Homolog Family Member A	RHOA
448	Cytochrome P450 Family 19 Subfamily A Member 1	CYP19A1
449	Parkinsonism Associated Deglycase	PARK7
450	NOC2 Like Nucleolar Associated Transcriptional Repressor	NOC2L
451	Matrix Metallopeptidase 12	MMP12
452	CD1a Molecule	CD1A
453	MN1 Proto-Oncogene, Transcriptional Regulator	MN1
454	Ectodysplasin A Receptor	EDAR
455	MicroRNA 30a	MIR30A
456	Neural Cell Adhesion Molecule 1	NCAM1
457	Cytochrome P450 Family 3 Subfamily A Member 4	CYP3A4
458	Neurotrophic Receptor Tyrosine Kinase 1	NTRK1
459	Cytochrome B5 Type A	CYB5A
460	RAB6B, Member RAS Oncogene Family	RAB6B
461	F-Box And Leucine Rich Repeat Protein 5	FBXL5
462	T Cell Receptor Beta Locus	TRB
463	Somatostatin	SST
464	Cytochrome P450 Family 2 Subfamily C Member 19	CYP2C19
465	MicroRNA 20a	MIR20A
466	CD2 Molecule	CD2
467	Delta Like Canonical Notch Ligand 1	DLL1
468	CD19 Molecule	CD19
469	Serpin Family H Member 1	SERPINH1
470	GBA Recombination Region	LOC106627981
471	Lysyl Oxidase	LOX
472	Mannosidase Alpha Class 2B Member 1	MAN2B1

473	Sialic Acid Binding Ig Like Lectin 5	SIGLEC5
474	Interleukin 1 Receptor Accessory Protein	IL1RAP
475	Signaling Lymphocytic Activation Molecule Family Member 1	SLAMF1
476	Transient Receptor Potential Cation Channel Subfamily A Member 1	TRPA1
477	Kruppel Like Factor 4	KLF4
478	RUNX Family Transcription Factor 1	RUNX1
479	Activin A Receptor Like Type 1	ACVRL1
480	Zinc Finger And BTB Domain Containing 20	ZBTB20
481	Bone Morphogenetic Protein 7	BMP7
482	Growth Arrest Specific 5	GAS5
483	ADAMTS Like 1	ADAMTSL1
484	Elongator Acetyltransferase Complex Subunit 1	ELP1
485	Cyclin Dependent Kinase Inhibitor 2C	CDKN2C
486	Ectodysplasin A	EDA
487	Transforming Growth Factor Alpha	TGFA
488	B Cell Scaffold Protein With Ankyrin Repeats 1	BANK1
489	Caspase 9	CASP9
490	Peptidyl Arginine Deiminase 4	PADI4
491	Poly(ADP-Ribose) Polymerase 1	PARP1
492	MutS Homolog 6	MSH6
493	Transient Receptor Potential Cation Channel Subfamily V Member 3	TRPV3
494	N-Acylsphingosine Amidohydrolase 1	ASAH1
495	Mitogen-Activated Protein Kinase Kinase 1	MAP2K1
496	Ectonucleotide Pyrophosphatase/Phosphodiesterase 1	ENPP1
497	Bone Morphogenetic Protein 4	BMP4
498	Cartilage Oligomeric Matrix Protein	COMP
499	Forkhead Box O1	FOXO1
500	Prolactin	PRL
501	Eukaryotic Translation Initiation Factor 1A Domain Containing	EIF1AD
502	Transient Receptor Potential Cation Channel Subfamily M Member 4	TRPM4
503	Sphingomyelin Phosphodiesterase 1	SMPD1
504	Exostosin Glycosyltransferase 2	EXT2
505	Ribosomal Protein S27a	RPS27A
506	Emerin	EMD
507	Centrosomal Protein 57	CEP57
508	BUB1 Mitotic Checkpoint Serine/Threonine Kinase B	BUB1B
509	Interleukin 17C	IL17C
510	Kinase Insert Domain Receptor	KDR
511	Cyclic GMP-AMP Synthase	CGAS
512	ADAM Metallopeptidase With Thrombospondin Type 1 Motif 3	ADAMTS3
513	Exostosin Glycosyltransferase 1	EXT1

514	P53 Apoptosis Effector Related To PMP22	PERP
515	Low Density Lipoprotein Receptor	LDLR
516	Inducible T Cell Costimulator	ICOS
517	Serine Protease 1	PRSS1
518	Hes Related Family BHLH Transcription Factor With YRPW Motif 2	HEY2
519	Mitogen-Activated Protein Kinase 10	MAPK10
520	Adaptor Related Protein Complex 1 Subunit Beta 1	AP1B1
521	MicroRNA 27a	MIR27A
522	Coproporphyrinogen Oxidase	CPOX
523	Solute Carrier Family 17 Member 5	SLC17A5
524	EGFR Antisense RNA 1	EGFR-AS1
525	MicroRNA 15a	MIR15A
526	Kallikrein Related Peptidase 3	KLK3
527	Myosin Heavy Chain 11	MYH11
528	S100 Calcium Binding Protein A7	S100A7
529	Prostaglandin E Receptor 1	PTGER1
530	Platelet Derived Growth Factor Subunit B	PDGFB
531	CD58 Molecule	CD58
532	Tenascin XB	TNXB
533	Filamin A	FLNA
534	BUB1 Mitotic Checkpoint Serine/Threonine Kinase	BUB1
535	Rho Associated Coiled-Coil Containing Protein Kinase 1	ROCK1
536	Nuclear Receptor Subfamily 5 Group A Member 2	NR5A2
537	Unc-45 Myosin Chaperone B	UNC45B
538	ERCC Excision Repair 1, Endonuclease Non-Catalytic Subunit	ERCC1
539	Platelet Derived Growth Factor Subunit A	PDGFA
540	ELAV Like RNA Binding Protein 1	ELAVL1
541	Fibroblast Growth Factor Receptor 2	FGFR2
542	Cathepsin L	CTSL
543	CD38 Molecule	CD38
544	Protein Tyrosine Phosphatase Non-Receptor Type 11	PTPN11
545	Serpin Family B Member 5	SERPINB5
546	Collagen Type V Alpha 1 Chain	COL5A1
547	Atypical Chemokine Receptor 1 (Duffy Blood Group)	ACKR1
548	Hepcidin Antimicrobial Peptide	HAMP
549	Apolipoprotein B	APOB
550	MAPK Associated Protein 1	MAPKAP1
551	Protein Tyrosine Kinase 2 Beta	PTK2B
552	MAGE Family Member F1	MAGEF1
553	Chymase 1	CMA1
554	Transglutaminase 1	TGM1
555	Heat Shock Protein Family A (Hsp70) Member 5	HSPA5
556	ASXL Transcriptional Regulator 1	ASXL1

557	Prosaposin	PSAP
558	Interleukin 34	IL34
559	Neurotrophic Receptor Tyrosine Kinase 3	NTRK3
560	T Cell Immune Regulator 1, ATPase H+ Transporting V0 Subunit A3	TCIRG1
561	Interferon Lambda 1	IFNL1
562	Hes Related Family BHLH Transcription Factor With YRPW Motif Like	HEYL
563	Cytochrome P450 Family 1 Subfamily A Member 2	CYP1A2
564	Alkaline Phosphatase, Placental	ALPP
565	MicroRNA 16-1	MIR16-1
566	Cell Division Cycle 73	CDC73
567	Cytochrome P450 Family 2 Subfamily E Member 1	CYP2E1
568	Prohibitin	PHB
569	Cytochrome P450 Family 1 Subfamily A Member 1	CYP1A1
570	Nuclear Factor Of Activated T Cells 1	NFATC1
571	BUB3 Mitotic Checkpoint Protein	BUB3
572	Chitinase Acidic	CHIA
573	Nuclear Paraspeckle Assembly Transcript 1	NEAT1
574	Prostaglandin E Receptor 3	PTGER3
575	SRY-Box Transcription Factor 2	SOX2
576	Leptin, Serum Levels Of	LEPQTL1
577	Syndecan 1	SDC1
578	CDK5 Regulatory Subunit Associated Protein 1 Like 1	CDKAL1
579	Integrin Subunit Alpha 6	ITGA6
580	Cbl Proto-Oncogene	CBL
581	Ectonucleotide Pyrophosphatase/Phosphodiesterase 3	ENPP3
582	ERCC Excision Repair 6, Chromatin Remodeling Factor	ERCC6
583	MicroRNA 200a	MIR200A
584	Protein Kinase, DNA-Activated, Catalytic Subunit	PRKDC
585	Adenosine A2b Receptor	ADORA2B
586	Sclerostin	SOST
587	Hes Family BHLH Transcription Factor 7	HES7
588	Lipopolysaccharide Induced TNF Factor	LITAF
589	MicroRNA 335	MIR335
590	Frizzled Related Protein	FRZB
591	Caspase Recruitment Domain Family Member 16	CARD16
592	Glutamate Ionotropic Receptor NMDA Type Subunit 2B	GRIN2B
593	Protein Kinase CAMP-Dependent Type I Regulatory Subunit Alpha	PRKAR1A
594	Peptidylprolyl Isomerase G	PPIG
595	Insulin Like Growth Factor 2	IGF2
596	Actin Alpha 2, Smooth Muscle	ACTA2
597	Integrin Subunit Beta 3	ITGB3

598	Jagged Canonical Notch Ligand 2	JAG2
599	Atlastin GTPase 3	ATL3
600	H19 Imprinted Maternally Expressed Transcript	H19
601	Chitinase 1	CHIT1
602	CDKN2B Antisense RNA 1	CDKN2B-AS1
603	Integrin Subunit Alpha V	ITGAV
604	TNF Superfamily Member 14	TNFSF14
605	Caldesmon 1	CALD1
606	Cathepsin D	CTSD
607	Basigin (Ok Blood Group)	BSG
608	Methylenetetrahydrofolate Dehydrogenase, Cyclohydrolase And Formyltetrahydrofolate Synthetase 1	MTHFD1
609	Growth Hormone 1	GH1
610	TAR DNA Binding Protein	TARDBP
611	Factor Interacting With PAPOLA And CPSF1	FIP1L1
612	RANBP2-Type And C3HC4-Type Zinc Finger Containing 1	RBCK1
613	Serpin Family F Member 1	SERPINF1
614	Lamin B2	LMNB2
615	ADAM Metallopeptidase With Thrombospondin Type 1 Motif 2	ADAMTS2
616	Loricrin Cornified Envelope Precursor Protein	LORICRIN
617	MutY DNA Glycosylase	MUTYH
618	Maternally Expressed 3	MEG3
619	Apolipoprotein D	APOD
620	Calcium Sensing Receptor	CASR
621	Cystatin E/M	CST6
622	Sphingosine-1-Phosphate Receptor 1	S1PR1
623	Thrombospondin 3	THBS3
624	Sterol Regulatory Element Binding Transcription Factor 2	SREBF2
625	Spectrin Repeat Containing Nuclear Envelope Protein 2	SYNE2
626	Membrane Spanning 4-Domains A1	MS4A1
627	Hyaluronan Synthase 1	HAS1
628	Serine And Arginine Rich Splicing Factor 2	SRSF2
629	Survival Of Motor Neuron 1, Telomeric	SMN1
630	CD1e Molecule	CD1E
631	Integrin Subunit Alpha 2	ITGA2
632	TNF Receptor Superfamily Member 13B	TNFRSF13B
633	CD47 Molecule	CD47
634	ATRX Chromatin Remodeler	ATRX
635	ERCC Excision Repair 4, Endonuclease Catalytic Subunit	ERCC4
636	C-Type Lectin Domain Containing 5A	CLEC5A
637	PAXIP1 Associated Glutamate Rich Protein 1	PAGR1
638	Collagen Type V Alpha 2 Chain	COL5A2
639	Transportin 1	TNPO1
640	Coagulation Factor IX	F9

641	Matrix Metallopeptidase 10	MMP10
642	Macrophage Stimulating 1 Receptor	MST1R
643	FKBP Prolyl Isomerase 14	FKBP14
644	Sirtuin 6	SIRT6
645	TNF Receptor Associated Factor 1	TRAF1
646	Transient Receptor Potential Cation Channel Subfamily V Member 2	TRPV2
647	ADAM Metallopeptidase With Thrombospondin Type 1 Motif 13	ADAMTS13
648	Ezrin	EZR
649	Inhibin Subunit Beta A	INHBA
650	Cytochrome P450 Family 3 Subfamily A Member 5	CYP3A5
651	Interferon Regulatory Factor 4	IRF4
652	MicroRNA 9-1	MIR9-1
653	SRY-Box Transcription Factor 4	SOX4
654	Solute Carrier Family 6 Member 19	SLC6A19
655	Fibulin 5	FBLN5
656	EGF Containing Fibulin Extracellular Matrix Protein 2	EFEMP2
657	Spi-1 Proto-Oncogene	SPI1
658	CD1d Molecule	CD1D
659	XPA, DNA Damage Recognition And Repair Factor	XPA
660	Thymidine Phosphorylase	TYMP
661	Sonic Hedgehog Signaling Molecule	SHH
662	Transducin Beta Like 1 X-Linked	TBL1X
663	MicroRNA 143	MIR143
664	Hyaluronan Synthase 2	HAS2
665	Choline Kinase Alpha	CHKA
666	Dickkopf WNT Signaling Pathway Inhibitor 1	DKK1
667	H2A.X Variant Histone	H2AX
668	TNF Superfamily Member 8	TNFSF8
669	B Cell Linker	BLNK
670	ERCC Excision Repair 8, CSA Ubiquitin Ligase Complex Subunit	ERCC8
671	BCL6 Transcription Repressor	BCL6
672	Decorin	DCN
673	Ganglioside Induced Differentiation Associated Protein 1	GDAP1
674	ERCC Excision Repair 2, TFIIH Core Complex Helicase Subunit	ERCC2
675	Hematopoietic Prostaglandin D Synthase	HPGDS
676	Succinate Dehydrogenase Complex Iron Sulfur Subunit B	SDHB
677	Periostin	POSTN
678	TXK Tyrosine Kinase	TXK
679	Cortactin	CTTN
680	Lymphocyte Antigen 75	LY75
681	ADAM Metallopeptidase Domain 12	ADAM12
682	SCN1A And SCN9A Antisense RNA 1	SCN1A-AS1
683	ADAM Metallopeptidase Domain 8	ADAM8

684	Late Cornified Envelope 3C	LCE3C
685	Late Cornified Envelope 3B	LCE3B
686	Synaptophysin	SYP
687	JunB Proto-Oncogene, AP-1 Transcription Factor Subunit	JUNB
688	Chromobox 5	CBX5
689	Galactosidase Alpha	GLA
690	Polypeptide N-Acetylgalactosaminyltransferase 3	GALNT3
691	Cadherin 11	CDH11
692	TNF Receptor Associated Factor 3	TRAF3
693	Matrix Metallopeptidase 25	MMP25
694	Fascin Actin-Bundling Protein 1	FSCN1
695	MicroRNA 30b	MIR30B
696	Differentiation Antagonizing Non-Protein Coding RNA	DANCR
697	Dihydrofolate Reductase	DHFR
698	Survival Of Motor Neuron 2, Centromeric	SMN2
699	Glutathione Synthetase	GSS
700	Mitochondrial Antiviral Signaling Protein	MAVS
701	EGF Containing Fibulin Extracellular Matrix Protein 1	EFEMP1
702	Integrin Binding Sialoprotein	IBSP
703	Ras Converting CAAX Endopeptidase 1	RCE1
704	Peptidyl Arginine Deiminase 2	PADI2
705	Pleckstrin	PLEK
706	Serine Palmitoyltransferase Long Chain Base Subunit 1	SPTLC1
707	ETS2 Repressor Factor	ERF
708	Peptidylprolyl Cis/Trans Isomerase, NIMA-Interacting 1	PIN1
709	FUS RNA Binding Protein	FUS
710	ATP Binding Cassette Subfamily C Member 9	ABCC9
711	Ceramide Synthase 3	CERS3
712	Gastrin	GAST
713	SRY-Box Transcription Factor 9	SOX9
714	Calcitonin Receptor	CALCR
715	Cullin 1	CUL1
716	C-Type Lectin Domain Family 4 Member E	CLEC4E
717	Placental Growth Factor	PGF
718	Snail Family Transcriptional Repressor 1	SNAI1
719	TIA1 Cytotoxic Granule Associated RNA Binding Protein	TIA1
720	Proline-Serine-Threonine Phosphatase Interacting Protein 2	PSTPIP2
721	Ubiquitination Factor E4A	UBE4A
722	CD70 Molecule	CD70
723	Immunoglobulin Heavy Constant Mu	IGHM
724	Dyskerin Pseudouridine Synthase 1	DKC1
725	Chromosome 1 Open Reading Frame 141	C1orf141
726	Hemoglobin Subunit Alpha 2	HBA2
727	Sirtuin 2	SIRT2

728	Furin, Paired Basic Amino Acid Cleaving Enzyme	FURIN
729	TGF-Beta Activated Kinase 1 (MAP3K7) Binding Protein 2	TAB2
730	ANTXR Cell Adhesion Molecule 1	ANTXR1
731	Transcription Factor 3	TCF3
732	Leucine Rich Repeat Containing 8 VRAC Subunit A	LRRC8A
733	CD79b Molecule	CD79B
734	Prostaglandin Reductase 1	PTGR1
735	E2F Transcription Factor 1	E2F1
736	Sad1 And UNC84 Domain Containing 2	SUN2
737	ADAM Metallopeptidase With Thrombospondin Type 1 Motif 9	ADAMTS9
738	Ring Finger Protein 1	RING1
739	Carbonic Anhydrase 2	CA2
740	Galactosylceramidase	GALC
741	Growth Hormone Receptor	GHR
742	Immunoglobulin Lambda Like Polypeptide 1	IGLL1
743	CXADR Ig-Like Cell Adhesion Molecule	CXADR
744	Apoptotic Peptidase Activating Factor 1	APAF1
745	Solute Carrier Family 29 Member 1 (Augustine Blood Group)	SLC29A1
746	Solute Carrier Family 2 Member 10	SLC2A10
747	ADAM Metallopeptidase Domain 28	ADAM28
748	HOX Transcript Antisense RNA	HOTAIR
749	TIMP Metallopeptidase Inhibitor 4	TIMP4
750	Phosphoribosyl Pyrophosphate Synthetase 1	PRPS1
751	GLIS Family Zinc Finger 3	GLIS3
752	Reticulon 4	RTN4
753	Double PHD Fingers 2	DPF2
754	Alkaline Phosphatase, Biomineralization Associated	ALPL
755	ADAM Metallopeptidase With Thrombospondin Type 1 Motif 6	ADAMTS6
756	Noggin	NOG
757	C-Type Lectin Domain Family 1 Member A	CLEC1A
758	Pvt1 Oncogene	PVT1
759	Fibroblast Growth Factor 23	FGF23
760	LIM Domain Kinase 1	LIMK1
761	Sphingomyelin Phosphodiesterase 3	SMPD3
762	Hexosaminidase Subunit Alpha	HEXA
763	Ankyrin 3	ANK3
764	Insulin Like Growth Factor Binding Protein 5	IGFBP5
765	Dentin Sialophosphoprotein	DSPP
766	Salt Inducible Kinase 2	SIK2
767	Lymphoid Enhancer Binding Factor 1	LEF1
768	Phospholipase A2 Activating Protein	PLAA
769	NRAS Proto-Oncogene, GTPase	NRAS
770	Integrin Subunit Alpha 1	ITGA1
771	MicroRNA 24-1	MIR24-1

772	Adenylate Cyclase 1	ADCY1
773	G Protein-Coupled Receptor 101	GPR101
774	ADAM Metallopeptidase With Thrombospondin Type 1 Motif 17	ADAMTS17
775	POU Class 2 Homeobox Associating Factor 1	POU2AF1
776	NCK Adaptor Protein 1	NCK1
777	N-Acetylneuraminic Acid Phosphatase	NANP
778	Golgin, RAB6 Interacting	GORAB
779	RUNX Family Transcription Factor 3	RUNX3
780	Ring Finger Protein 31	RNF31
781	Parkin RBR E3 Ubiquitin Protein Ligase	PRKN
782	N-Sulfoglucosamine Sulfohydrolase	SGSH
783	Growth Hormone Releasing Hormone	GHRH
784	SHANK Associated RH Domain Interactor	SHARPIN
785	MAF BZIP Transcription Factor	MAF
786	CD1c Molecule	CD1C
787	Dopamine Beta-Hydroxylase	DBH
788	SHC Adaptor Protein 1	SHC1
789	Gap Junction Protein Beta 3	GJB3
790	C-Type Lectin Domain Family 4 Member D	CLEC4D
791	WASP Like Actin Nucleation Promoting Factor	WASL
792	Maltase-Glucoamylase	MGAM
793	Melanocyte Inducing Transcription Factor	MITF
794	Solute Carrier Family 19 Member 1	SLC19A1
795	Matrix Gla Protein	MGP
796	CD1b Molecule	CD1B
797	Aminomethyltransferase	AMT
798	ATPase Copper Transporting Beta	ATP7B
799	Transient Receptor Potential Cation Channel Subfamily M Member 8	TRPM8
800	Collagen Type VI Alpha 5 Chain	COL6A5
801	Nuclear Receptor Subfamily 1 Group I Member 3	NR1I3
802	Bone Morphogenetic Protein Receptor Type 2	BMPR2
803	Cell Migration Inducing Hyaluronidase 1	CEMIP
804	Solute Carrier Family 29 Member 2	SLC29A2
805	Fms Related Receptor Tyrosine Kinase 3	FLT3
806	FLVCR Heme Transporter 1	FLVCR1
807	Ferrochelatase	FECH
808	Aryl Hydrocarbon Receptor Interacting Protein	AIP
809	Ubiquitin Recognition Factor In ER Associated Degradation 1	UFD1
810	TNF Receptor Superfamily Member 10d	TNFRSF10D
811	Calcium Activated Nucleotidase 1	CANT1
812	Cadherin 3	CDH3
813	RAN, Member RAS Oncogene Family	RAN
814	Solute Carrier Family 22 Member 2	SLC22A2

815	Sp7 Transcription Factor	SP7
816	Glycoprotein Nmb	GPNMB
817	Cbp/P300 Interacting Transactivator With Glu/Asp Rich Carboxy-Terminal Domain 2	CITED2
818	Rhomboid 5 Homolog 2	RHBDF2
819	Tec Protein Tyrosine Kinase	TEC
820	Spectrin Repeat Containing Nuclear Envelope Protein 1	SYNE1
821	Protein Phosphatase 2 Phosphatase Activator	PTPA
822	Chaperonin Containing TCP1 Subunit 5	CCT5
823	Lectin, Mannose Binding 1	LMAN1
824	Paired Box 5	PAX5
825	Ceramide Kinase	CERK
826	Succinate Dehydrogenase Complex Subunit C	SDHC
827	Gap Junction Protein Beta 4	GJB4
828	LMBR1 Domain Containing 1	LMBRD1
829	Dynein Axonemal Assembly Factor 2	DNAAF2
830	WRN RecQ Like Helicase	WRN
831	Matrix Metallopeptidase 11	MMP11
832	Spectrin Repeat Containing Nuclear Envelope Family Member 3	SYNE3
833	Prostate Cancer Associated Non-Coding RNA 1	PRNCR1
834	JunD Proto-Oncogene, AP-1 Transcription Factor Subunit	JUND
835	Chloride Voltage-Gated Channel 7	CLCN7
836	Pleckstrin Homology And RUN Domain Containing M1	PLEKHM1
837	Neuropilin 1	NRP1
838	Apoptosis Inducing Factor Mitochondria Associated 1	AIFM1
839	Neurotrophic Receptor Tyrosine Kinase 2	NTRK2
840	Osteoclastogenesis Associated Transmembrane Protein 1	OSTM1
841	Transient Receptor Potential Cation Channel Subfamily C Member 6	TRPC6
842	ADAM Metallopeptidase Domain 9	ADAM9
843	RAB7A, Member RAS Oncogene Family	RAB7A
844	Mitochondrially Encoded Cytochrome B	MT-CYB
845	Twist Family BHLH Transcription Factor 2	TWIST2
846	DnaJ Heat Shock Protein Family (Hsp40) Member B11	DNAJB11
847	Tumor Protein P53 Binding Protein 1	TP53BP1
848	Polo Like Kinase 1	PLK1
849	Lysosomal Associated Membrane Protein 3	LAMP3
850	Calbindin 2	CALB2
851	MicroRNA 377	MIR377
852	Urothelial Cancer Associated 1	UCA1
853	Semaphorin 3A	SEMA3A
854	Succinate Dehydrogenase Complex Subunit D	SDHD
855	Interleukin 3 Receptor Subunit Alpha	IL3RA
856	X Inactive Specific Transcript	XIST

857	Transforming Growth Factor Beta Induced	TGFBI
858	ETS Variant Transcription Factor 6	ETV6
859	T-Box Transcription Factor 3	TBX3
860	Filamin Binding LIM Protein 1	FBLIM1
861	Solute Carrier Family 12 Member 2	SLC12A2
862	Solute Carrier Family 7 Member 11	SLC7A11
863	BSCL2 Lipid Droplet Biogenesis Associated, Seipin	BSCL2
864	Serpin Family B Member 4	SERPINB4
865	Serine Palmitoyltransferase Long Chain Base Subunit 2	SPTLC2
866	Colon Cancer Associated Transcript 1	CCAT1
867	Leucine Rich Repeat Containing G Protein-Coupled Receptor 5	LGR5
868	Transmembrane Protein 178A	TMEM178A
869	Endoplasmic Reticulum-Golgi Intermediate Compartment 1	ERGIC1
870	MicroRNA 186	MIR186
871	Bone Morphogenetic Protein Receptor Type 1A	BMPR1A
872	Cyclin Dependent Kinase 6	CDK6
873	SH3 Domain And Tetratricopeptide Repeats 2	SH3TC2
874	MicroRNA 29b-1	MIR29B1
875	Taurine Up-Regulated 1	TUG1
876	Stabilin 1	STAB1
877	Lysophosphatidic Acid Receptor 3	LPAR3
878	LSM2 Homolog, U6 Small Nuclear RNA And MRNA Degradation Associated	LSM2
879	MicroRNA 211	MIR211
880	G Protein Subunit Alpha 12	GNA12
881	Solute Carrier Organic Anion Transporter Family Member 4A1	SLCO4A1
882	PC4 And SFRS1 Interacting Protein 1	PSIP1
883	Mitochondrial Ribosomal Protein L45	MRPL45
884	Rho GTPase Activating Protein 23	ARHGAP23
885	Phospholipase B1	PLB1
886	Serine/Threonine Kinase 39	STK39
887	Laminin Subunit Alpha 1	LAMA1
888	Aldo-Keto Reductase Family 1 Member C2	AKR1C2
889	SWI/SNF Related, Matrix Associated, Actin Dependent Regulator Of Chromatin, Subfamily D, Member 2	SMARCD2
890	ATPase Family AAA Domain Containing 1	ATAD1
891	Adenosine Deaminase TRNA Specific 3	ADAT3
892	Beta-1,3-Glucuronyltransferase 1	B3GAT1
893	2'-5'-Oligoadenylate Synthetase 1	OAS1
894	Iodothyronine Deiodinase 2	DIO2
895	Pericentrin	PCNT
896	Transient Receptor Potential Cation Channel Subfamily M Member 7	TRPM7
897	Growth Hormone Releasing Hormone Receptor	GHRHR

898	Replication Factor C Subunit 1	RFC1
899	Dopachrome Tautomerase	DCT
900	Lysosomal Associated Membrane Protein 2	LAMP2
901	ABL Proto-Oncogene 1, Non-Receptor Tyrosine Kinase	ABL1
902	Biglycan	BGN
903	Catenin Alpha 1	CTNNA1
904	Lysosomal Associated Membrane Protein 1	LAMP1
905	SPG7 Matrix AAA Peptidase Subunit, Paraplegin	SPG7
906	Ubiquitin Like Modifier Activating Enzyme 7	UBA7
907	BMX Non-Receptor Tyrosine Kinase	BMX
908	Myomesin 2	MYOM2
909	Long Intergenic Non-Protein Coding RNA 1554	LINC01554
910	Solute Carrier Family 40 Member 1	SLC40A1
911	Isoprenylcysteine Carboxyl Methyltransferase	ICMT
912	Cadherin 2	CDH2
913	CCDC26 Long Non-Coding RNA	CCDC26
914	Secreted LY6/PLAUR Domain Containing 1	SLURP1
915	Latent Transforming Growth Factor Beta Binding Protein 4	LTBP4
916	MicroRNA 374a	MIR374A
917	Dynein Cytoplasmic 2 Heavy Chain 1	DYNC2H1
918	S-Phase Kinase Associated Protein 1	SKP1
919	Nucleoporin 153	NUP153
920	Sad1 And UNC84 Domain Containing 1	SUN1
921	Glycerol-3-Phosphate Dehydrogenase 1	GPD1
922	Promoter Of CDKN1A Antisense DNA Damage Activated RNA	PANDAR
923	Secreted Frizzled Related Protein 1	SFRP1
924	Exportin 1	XPO1
925	Bone Marrow Stromal Cell Antigen 2	BST2
926	RNA Binding Region (RNP1, RRM) Containing 3	RNPC3
927	F-Box And WD Repeat Domain Containing 7	FBXW7
928	LDL Receptor Related Protein 5	LRP5
929	Immunoglobulin Mu DNA Binding Protein 2	IGHMBP2
930	Tubulin Alpha 1b	TUBA1B
931	Solute Carrier Family 25 Member 24	SLC25A24
932	Senataxin	SETX
933	Solute Carrier Family 12 Member 5	SLC12A5
934	Hepatocellular Carcinoma Up-Regulated Long Non-Coding RNA	HULC
935	HOXA Distal Transcript Antisense RNA	HOTTIP
936	PTEN Induced Kinase 1	PINK1
937	TBC1 Domain Family Member 3	TBC1D3
938	Dehydrogenase/Reductase 2	DHRS2
939	Death Domain Associated Protein	DAXX
940	Cyclin G1	CCNG1
941	N-Acylsphingosine Amidohydrolase 2	ASAH2

942	U2 Small Nuclear RNA Auxiliary Factor 1	U2AF1
943	Pyruvate Kinase L/R	PKLR
944	Nuclear Factor Of Activated T Cells 3	NFATC3
945	Integrin Linked Kinase	ILK
946	Angiogenic Factor With G-Patch And FHA Domains 1	AGGF1
947	Sphingomyelin Phosphodiesterase Acid Like 3B	SMPDL3B
948	V-Set And Immunoglobulin Domain Containing 4	VSIG4
949	ATPase H+ Transporting Accessory Protein 1	ATP6AP1
950	Actin Filament Associated Protein 1	AFAP1
951	Glycophorin C (Gerbich Blood Group)	GYPC
952	Inhibitor Of DNA Binding 1, HLH Protein	ID1
953	ZFP57 Zinc Finger Protein	ZFP57
954	SH3 And PX Domains 2A	SH3PXD2A
955	SUMO Specific Peptidase 7	SENP7
956	Arylsulfatase A	ARSA
957	Lipase A, Lysosomal Acid Type	LIPA
958	C-Type Lectin Domain Containing 6A	CLEC6A
959	Cell Division Cycle 20	CDC20
960	Uncharacterized LOC100287329	LOC100287329
961	Pyruvate Dehydrogenase Phosphatase Catalytic Subunit 1	PDP1
962	T-Box Transcription Factor 5	TBX5
963	Antioxidant 1 Copper Chaperone	ATOX1
964	UDP-Glucose Ceramide Glucosyltransferase	UGCG
965	MicroRNA 483	MIR483
966	Mannosidase Beta	MANBA
967	Catenin Alpha 2	CTNNA2
968	Mannosidase Alpha Class 1B Member 1	MAN1B1
969	MacroH2A.1 Histone	MACROH2A1
970	Regulator Of G Protein Signaling 5	RGS5
971	Integrin Subunit Beta 5	ITGB5
972	Proprotein Convertase Subtilisin/Kexin Type 6	PCSK6
973	WT1 Transcription Factor	WT1
974	Von Hippel-Lindau Tumor Suppressor	VHL
975	Cathepsin E	CTSE
976	Zinc Finger Protein 683	ZNF683
977	Phospholipase C Beta 3	PLCB3
978	Ubiquitination Factor E4B	UBE4B
979	HOXA11 Antisense RNA	HOXA11-AS
980	Solute Carrier Family 28 Member 3	SLC28A3
981	MIR210 Host Gene	MIR210HG
982	Minichromosome Maintenance Complex Component 5	MCM5
983	NPC Intracellular Cholesterol Transporter 2	NPC2
984	Peptidylglycine Alpha-Amidating Monooxygenase	PAM
985	Bone Morphogenetic Protein 3	BMP3

986	C9orf72-SMCR8 Complex Subunit	C9orf72
987	Heat Shock Protein Family B (Small) Member 8	HSPB8
988	RNA Binding Motif Protein 19	RBM19
989	Wnt Family Member 3A	WNT3A
990	Keratin 15	KRT15
991	WSC Domain Containing 2	WSCD2
992	Scm Like With Four Mbt Domains 2	SFMBT2
993	Small Nuclear Ribonucleoprotein U1 Subunit 70	SNRNP70
994	Cancer Susceptibility 2	CASC2
995	Alpha-L-Iduronidase	IDUA
996	DEF6 Guanine Nucleotide Exchange Factor	DEF6
997	Immunoglobulin Heavy Constant Gamma 3 (G3m Marker)	IGHG3
998	FAU Ubiquitin Like And Ribosomal Protein S30 Fusion	FAU
999	WW Domain Containing Transcription Regulator 1	WWTR1
1000	Zinc Finger FYVE-Type Containing 16	ZFYVE16
1001	MicroRNA 342	MIR342
1002	Cytochrome P450 Family 51 Subfamily A Member 1	CYP51A1
1003	Heterogeneous Nuclear Ribonucleoprotein C	HNRNPC
1004	RING1 And YY1 Binding Protein	RYBP
1005	MicroRNA 18a	MIR18A
1006	ST8 Alpha-N-Acetyl-Neuraminide Alpha-2,8-Sialyltransferase 4	ST8SIA4
1007	Transient Receptor Potential Cation Channel Subfamily M Member 3	TRPM3
1008	Solute Carrier Family 34 Member 1	SLC34A1
1009	Mastermind Like Transcriptional Coactivator 3	MAML3
1010	Secreted Frizzled Related Protein 2	SFRP2
1011	ATPase H+ Transporting V0 Subunit A2	ATP6V0A2
1012	Insulin Like Growth Factor Binding Protein 6	IGFBP6
1013	Small Nuclear Ribonucleoprotein Polypeptide N	SNRPN
1014	Bone Morphogenetic Protein 1	BMP1
1015	Leucine Rich Repeat Kinase 1	LRRK1
1016	Heterogeneous Nuclear Ribonucleoprotein D Like	HNRNPDL
1017	Serpin Family B Member 3	SERPINB3
1018	Ring-Box 1	RBX1
1019	Anti-Mullerian Hormone	AMH
1020	Vav Guanine Nucleotide Exchange Factor 3	VAV3
1021	Radical S-Adenosyl Methionine Domain Containing 2	RSAD2
1022	Glycogen Phosphorylase, Muscle Associated	PYGM
1023	OCA2 Melanosomal Transmembrane Protein	OCA2
1024	WW Domain Containing E3 Ubiquitin Protein Ligase 1	WWP1
1025	Macrophage Receptor With Collagenous Structure	MARCO
1026	Ataxin 3	ATXN3
1027	Leucine Zipper Tumor Suppressor 1	LZTS1
1028	Transcription Factor EB	TFEB

1029	ATP Binding Cassette Subfamily C Member 5	ABCC5
1030	Complement Factor H Related 1	CFHR1
1031	Zinc Finger DHHC-Type Palmitoyltransferase 2	ZDHHC2
1032	Cystatin F	CST7
1033	PTGS2 Antisense NFKB1 Complex-Mediated Expression Regulator RNA	PACERR
1034	Protoporphyrinogen Oxidase	PPOX
1035	D-2-Hydroxyglutarate Dehydrogenase	D2HGDH
1036	Alpha And Gamma Adaptin Binding Protein	AAGAB
1037	Metaxin 1	MTX1
1038	Mannosidase Alpha Class 2C Member 1	MAN2C1
1039	Centrosomal Protein 89	CEP89
1040	Centrosomal Protein 192	CEP192
1041	DnaJ Heat Shock Protein Family (Hsp40) Member C27	DNAJC27
1042	Meiotic Double-Stranded Break Formation Protein 1	MEI1
1043	Prickle Planar Cell Polarity Protein 4	PRICKLE4
1044	Matrix Remodeling Associated 8	MXRA8
1045	Elongator Acetyltransferase Complex Subunit 6	ELP6
1046	Valosin Containing Protein Lysine Methyltransferase	VCPKMT
1047	HNF1A Antisense RNA 1	HNF1A-AS1
1048	Phosphodiesterase 11A	PDE11A
1049	Heterogeneous Nuclear Ribonucleoprotein H2	HNRNPH2
1050	Small Nuclear Ribonucleoprotein D3 Polypeptide	SNRPD3
1051	IKBKG Recombination Region	LOC107988021
1052	IKBKG Downstream Recombination Region	LOC107988022
1053	Amylo-Alpha-1, 6-Glucosidase, 4-Alpha-Glucanotransferase	AGL
1054	Uncharacterized LOC101448202	LOC101448202
1055	Deoxyhypusine Synthase	DHPS
1056	Syntaxin 5	STX5
1057	4-Hydroxyphenylpyruvate Dioxygenase	HPD
1058	Eukaryotic Translation Elongation Factor 1 Alpha 1	EEF1A1
1059	Syntrophin Beta 2	SNTB2
1060	MicroRNA 664a	MIR664A
1061	EGF Domain Specific O-Linked N-Acetylglucosamine Transferase	EOGT
1062	Small Nuclear Ribonucleoprotein Polypeptide A	SNRPA
1063	G Protein-Coupled Receptor 108	GPR108
1064	VPS35 Retromer Complex Component	VPS35
1065	Sphingomyelin Synthase 1	SGMS1
1066	COP9 Signalosome Subunit 3	COPS3
1067	Copper Metabolism Domain Containing 1	COMMD1
1068	Mannose-6-Phosphate Receptor, Cation Dependent	M6PR
1069	MicroRNA 139	MIR139
1070	Wnt Family Member 10B	WNT10B

1071	High Mobility Group AT-Hook 2	HMGA2
1072	Indian Hedgehog Signaling Molecule	IHH
1073	BRAF-Activated Non-Protein Coding RNA	BANCR
1074	Long Noncoding RNA Activated By TGF-Beta	LNCRNA-ATB
1075	Alpha-L-Fucosidase 1	FUCA1
1076	Centromere Protein E	CENPE
1077	Aquarius Intron-Binding Spliceosomal Factor	AQR
1078	Hemoglobin Subunit Theta 1	HBQ1
1079	Zinc Finger And BTB Domain Containing 6	ZBTB6
1080	Trafficking Protein Particle Complex Subunit 2B	TRAPPC2B
1081	Protein Tyrosine Phosphatase Receptor Type G	PTPRG
1082	Polycomb Group Ring Finger 2	PCGF2
1083	Mature T Cell Proliferation 1	MTCP1
1084	MicroRNA 614	MIR614
1085	MicroRNA 520a	MIR520A
1086	Colon Cancer Associated Transcript 2	CCAT2
1087	Myosin Heavy Chain Gene Cluster Antisense RNA	MYHAS
1088	IKBKGP1 Recombination Region	LOC107988024
1089	IKBKGP1 Upstream Recombination Region	LOC107988025
1090	Phytanoyl-CoA 2-Hydroxylase	PHYH
1091	5'-Aminolevulinate Synthase 1	ALAS1
1092	Transient Receptor Potential Cation Channel Subfamily C Member 3	TRPC3
1093	ZEB1 Antisense RNA 1	ZEB1-AS1
1094	Transient Receptor Potential Cation Channel Subfamily C Member 4	TRPC4
1095	Protein Tyrosine Kinase 6	PTK6
1096	Transient Receptor Potential Cation Channel Subfamily V Member 6	TRPV6
1097	Hydroxymethylbilane Synthase	HMBS
1098	Keratin 24	KRT24
1099	FOXF1 Adjacent Non-Coding Developmental Regulatory RNA	FENDRR
1100	NPC Intracellular Cholesterol Transporter 1	NPC1
1101	Farnesyl Diphosphate Synthase	FDPS
1102	Plexin A1	PLXNA1
1103	Golgi Reassembly Stacking Protein 1	GORASP1
1104	Solute Carrier Organic Anion Transporter Family Member 3A1	SLCO3A1
1105	Centromere Protein V	CENPV
1106	Small Nucleolar RNA Host Gene 1	SNHG1
1107	Prostate Cancer Associated Transcript 1	PCAT1
1108	NNT Antisense RNA 1	NNT-AS1
1109	G Protein Subunit Alpha 15	GNA15
1110	Mannose Receptor C Type 2	MRC2
1111	Sphingomyelin Phosphodiesterase 2	SMPD2

1112	TATA-Box Binding Protein Associated Factor 3	TAF3
1113	Matrix Metallopeptidase 16	MMP16
1114	Transcription Factor Binding To IGHM Enhancer 3	TFE3
1115	Nudix Hydrolase 6	NUDT6
1116	Fumarate Hydratase	FH
1117	Hemojuvelin BMP Co-Receptor	HJV
1118	Microtubule Affinity Regulating Kinase 3	MARK3
1119	Mannosidase Alpha Class 2A Member 1	MAN2A1
1120	Rho Related BTB Domain Containing 2	RHOBTB2
1121	Fibroblast Growth Factor 6	FGF6
1122	Uroplakin 1A	UPK1A
1123	Trichohyalin	TCHH
1124	SOX2 Overlapping Transcript	SOX2-OT
1125	Breast Cancer Anti-Estrogen Resistance 4	BCAR4
1126	HIF1A Antisense RNA 2	HIF1A-AS2
1127	FOXC2 Antisense RNA 1	FOXC2-AS1
1128	Long Intergenic Non-Protein Coding RNA 901	LINC00901
1129	Immunoglobulin Heavy Variable 4-38-2	IGHV4-38-2
1130	Ubiquitin Specific Peptidase 6	USP6
1131	WEE1 G2 Checkpoint Kinase	WEE1
1132	E2F Transcription Factor 4	E2F4
1133	Glucosylceramidase Beta 2	GBA2
1134	Golgin B1	GOLGB1
1135	PHD Finger Protein 6	PHF6
1136	SEC61 Translocon Subunit Alpha 1	SEC61A1
1137	Msh Homeobox 2	MSX2
1138	Transferrin Receptor 2	TFR2
1139	Ring Finger Protein 135	RNF135
1140	RB Transcriptional Corepressor Like 1	RBL1
1141	Phosphatidylinositol Transfer Protein Alpha	PITPNA
1142	Transient Receptor Potential Cation Channel Subfamily C Member 5	TRPC5
1143	Solute Carrier Family 15 Member 2	SLC15A2
1144	WW Domain Containing E3 Ubiquitin Protein Ligase 2	WWP2
1145	Phosphate Regulating Endopeptidase Homolog X-Linked	PHEX
1146	Diacylglycerol Kinase Theta	DGKQ
1147	IQ Motif Containing GTPase Activating Protein 2	IQGAP2
1148	G3BP Stress Granule Assembly Factor 1	G3BP1
1149	Smoothelin	SMTN
1150	RAB7B, Member RAS Oncogene Family	RAB7B
1151	CLN6 Transmembrane ER Protein	CLN6
1152	Mannose-Binding Lectin Family Member 3, Pseudogene	MBL3P
1153	Karyopherin Subunit Alpha 4	KPNA4
1154	2'-5'-Oligoadenylate Synthetase 3	OAS3

1155	UTP6 Small Subunit Processome Component	UTP6
1156	H3.3 Histone B	H3-3B
1157	Ephrin A2	EFNA2
1158	Olfactory Receptor Family 4 Subfamily D Member 2	OR4D2
1159	Dynactin Subunit 1	DCTN1
1160	Achaete-Scute Family BHLH Transcription Factor 1	ASCL1
1161	Calnexin	CANX
1162	Pancreatic Polypeptide	PPY
1163	G3BP Stress Granule Assembly Factor 2	G3BP2
1164	YEATS Domain Containing 2	YEATS2
1165	Melanotransferrin	MELTF
1166	Spartin	SPART
1167	Pancreatic And Duodenal Homeobox 1	PDX1
1168	Pyrroline-5-Carboxylate Reductase 1	PYCR1
1169	Aminolevulinate Dehydratase	ALAD
1170	Isocitrate Dehydrogenase (NAD(+)) 3 Non-Catalytic Subunit Beta	IDH3B
1171	Transient Receptor Potential Cation Channel Subfamily M Member 6	TRPM6
1172	FYVE, RhoGEF And PH Domain Containing 4	FGD4
1173	RB Transcriptional Corepressor Like 2	RBL2
1174	Ubiquilin 2	UBQLN2
1175	Golgin A2	GOLGA2
1176	Glutathione S-Transferase Mu 5	GSTM5
1177	Collectin Subfamily Member 12	COLEC12
1178	Ly1 Antibody Reactive	LYAR
1179	Zinc Fingers And Homeoboxes 2	ZHX2
1180	Centriolar Coiled-Coil Protein 110	CCP110
1181	Centlein	CNTLN
1182	Keratin 31	KRT31
1183	Coordinator Of PRMT5 And Differentiation Stimulator	COPRS
1184	Integrin Subunit Beta Like 1	ITGBL1
1185	Solute Carrier Family 12 Member 6	SLC12A6
1186	Aldehyde Dehydrogenase 18 Family Member A1	ALDH18A1
1187	5'-Aminolevulinate Synthase 2	ALAS2
1188	Glycophorin A (MNS Blood Group)	GYPA
1189	WNK Lysine Deficient Protein Kinase 4	WNK4
1190	Distal-Less Homeobox 3	DLX3
1191	Homeobox C13	HOXC13
1192	Secretion Associated Ras Related GTPase 1A	SAR1A
1193	Alpha-L-Fucosidase 2	FUCA2
1194	Tubulin Gamma Complex Associated Protein 6	TUBGCP6
1195	NDRG Family Member 4	NDRG4
1196	Acyl-CoA Synthetase Short Chain Family Member 3	ACSS3
1197	Lysine Methyltransferase 2B	KMT2B

1198	NPL4 Homolog, Ubiquitin Recognition Factor	NPLOC4
1199	RB Binding Protein 5, Histone Lysine Methyltransferase Complex Subunit	RBBP5
1200	NSF Attachment Protein Gamma	NAPG
1201	Pleckstrin Homology Like Domain Family B Member 1	PHLDB1
1202	Succinyl-CoA:Glutarate-CoA Transferase	SUGCT
1203	Keratin 72	KRT72
1204	Seryl-TRNA Synthetase 1	SARS1
1205	ATPase Family AAA Domain Containing 5	ATAD5
1206	Stress Induced Phosphoprotein 1	STIP1
1207	Dynein Light Chain LC8-Type 1	DYNLL1
1208	Placenta Associated 8	PLAC8
1209	Myeloma Overexpressed	MYEOV
1210	Farnesyl-Diphosphate Farnesyltransferase 1	FDFT1
1211	Osteoglycin	OGN
1212	H3.3 Histone A	H3-3A
1213	MicroRNA 218-1	MIR218-1
1214	DLC1 Rho GTPase Activating Protein	DLC1
1215	Nuclear Factor I B	NFIB
1216	EvC Ciliary Complex Subunit 1	EVC
1217	MicroRNA 218-2	MIR218-2
1218	Acid Phosphatase 2, Lysosomal	ACP2
1219	EvC Ciliary Complex Subunit 2	EVC2
